# Locking solutions for prevention of central venous access device complications in the adult critical care population: A systematic review

**DOI:** 10.1371/journal.pone.0289938

**Published:** 2023-10-12

**Authors:** Marlena Ornowska, Joshua Smithman, Steven Reynolds

**Affiliations:** 1 Department of Biomedical Physiology and Kinesiology, Simon Fraser University, Burnaby, BC, Canada; 2 Department of Biology, Simon Fraser University, Burnaby, BC, Canada; 3 Fraser Health Authority, Royal Columbian Hospital, New Westminster, BC, Canada; Ataturk University Faculty of Medicine, TURKEY

## Abstract

**Background:**

The objective of this systematic review is to determine the extent and quality of evidence for use of different types of locking fluids to prevent central venous access device complications in adult critical care patients. Specifically, rates of catheter-related bloodstream infection, colonization, and occlusion were considered. All types of devices were included in the review: central venous catheters, peripherally- inserted central catheters and hemodialysis catheters.

**Methods:**

**Eligibility criteria.** Papers had to include adult (>18 years old) critical care patients, be experimental trials, conducted in North America and Europe, and published in peer-reviewed journals from 2010 onwards.

**Information sources.** A search of Medline and EMBASE databases was performed. The search is current as of November 28^th^, 2022.

**Risk of bias.** The Cochrane Risk of Bias 2 and the Risk of Bias In Non-Randomized Studies of Intervention tools were used to assess the risk of bias in included studies.

**Results:**

**Included studies.** A total of 240 paper titles and abstracts underwent review, of these seven studies met the final criteria for quality appraisal. A total of three studies earned a low risk of bias quality appraisal.

**Discussion:**

**Limitations of evidence.** Due to heterogeneity of types of locking fluids investigated and small number of studies identified, meta-analysis of results was not possible.

**Interpretation.** Out of all fluids investigated, only citrate 46.7% was found to statistically reduce central venous access device complication rates. This systematic review has also identified a gap in the literature regarding studies of locking fluids that are adequately powered in this patient population.

**Future directions:**

Future research should include investigations and use of novel locking fluids with more effective properties against complications. It is imperative that future studies are adequately powered, randomized controlled trials in this patient population to facilitate optimal evidence-based care.

## Introduction

### Rationale

Central Venous Access Devices (CVADs) are intravenous catheters that are inserted into one of the centrally-located veins. Large veins such as the subclavian, internal jugular, and femoral veins are common insertion sites. The purpose of the CVAD is to administer large volumes of medications, fluids, or parenteral nutrition, and to easily access and draw a patient’s blood. Insertion of a CVAD also serves to protect smaller, peripheral veins from damage caused by highly irritant medications. Specialized CVADs may also be temporarily inserted to facilitate hemodialysis. As such, CVADs are commonly used in Intensive Care Units (ICUs) and High Acuity Units (HAUs), due to the advanced care needs of the critically ill. Types of CVADs most commonly used in this population include central venous catheters (CVCs), hemodialysis (HD) catheters, and peripherally-inserted central catheters (PICCs) [1].

Although an essential component of a patient’s treatment, the use of a CVAD is associated with a risk of complications. A CVAD can become infected when bacteria infiltrate the exterior of the catheter or the inside of the lumen. Once bacteria adhere to the catheter surface, they may develop into self-sustaining biofilm colonies with additional resistance to antibiotics [2]. Microbiological testing has shown biofilm growth to be present in up to 100% of CVADs, on both extraluminal and intraluminal surfaces [3]. A more recent study by Medis et al. has shown 97% of coagulase-negative *Staphylococcus* species isolated from CVCs of ICU patients to be strong biofilm formers with resistance to erythromycin, cloxacillin, clindamycin, and ciproflaxin [4]. These biofilm based bacteria may also spread into the bloodstream and result in difficult to treat and costly catheter-related bloodstream infections (CRBSIs). It is estimated that the odds of in-hospital mortality are almost 3 times greater than patients without central line-associated bloodstream infection [5]. In addition to complicating patient care, one episode of CRBSI is estimated to cost the US healthcare system up to $44,000 per case in the adult ICU setting [6].

The formation of bacterial biofilm along catheter surfaces also increases the risk of CVAD occlusion and thrombus formation. The biofilm inside a catheter lumen may be exposed to a variety of substances infused through the catheter such as crystalloid solutions, various drugs, nutrition, and blood or blood products. Particles of these substances may become incorporated into the biofilm and serve to occlude the CVAD, leading to interruption of treatment and the potential need for catheter replacement [2, 7, 8]. CVAD occlusion is quite common and affects approximately 22% of CVADs in the ICU setting [9]. The presence of foreign material in the bloodstream may also elicit an immune response cumulating in the activation of the coagulation cascade and the development of a thrombus, leading to further adverse events [2, 9, 10].

When a patient does not need a constant infusion of medication, nurses fill the inside of the CVAD with fluid to keep the inner lumen patent. This is called ‘locking’ the CVAD. When placed inside the catheter, this fluid prevents blood from filling and clotting inside, in the absence of flow through the lumen. As the locking fluid has access to the inside of the CVAD lumen for extended periods of time, there is therapeutic potential to utilize a fluid with antimicrobial, antibiofilm, and antithrombotic or anticoagulant properties to prevent the formation of catheter complications. Examples of locking fluids previously investigated in the adult critical care population include saline, heparin, ethanol, and sodium citrate.

### Objectives

The current standard of care in North America includes the use of saline for CVCs and PICCs, or 4% citrate for locking hemodialysis catheters in the critical care population [11, 12]. Heparin may also be used [12]. Though these fluids provide some protection from complications, CRBSI, CVAD colonization, and occlusion remain a problem. There is evidence from studies conducted in other patient populations of the effectiveness of other types of locking fluids in preventing the development of complications, such as antibiotic lock therapy, taurolidine, EDTA, or various combination products [13–17]. Despite this, current national guidelines indicate the use of these ‘alternative’ locking fluids only for patients with a history of CVAD-related infection, those with recently implanted cardiac devices, or those with long-term CVADs [11, 12]. Specifically in the case of non-tunnelled central venous catheters (which are inserted most commonly among the critical care population), expert consensus highlights the need for further investigation to support evidence-based locking guidelines using alternative fluids [18].

The purpose of this systematic review is to determine the extent and strength of evidence currently available for the use of various locking solutions in preventing CVAD complications in the adult critical care population. While locking solutions have been the subject of many systematic reviews and meta-analyses [19–27], these reviews focus only on one type of locking solution or one type of CVAD (often HD catheters), and are not specific to adult ICU/HAU patients. To our knowledge, this is the first review taking into consideration all types of locking fluids, and all types of CVADs in this specific population. The reporting of this systematic review was guided by the standards of the Preferred Reporting Items for Systematic Review and Meta-Analysis (PRISMA) Statement.

## Materials and methods

### Eligibility criteria

#### Inclusion criteria

Studies were selected to be part of the systematic review if they included adult (>18 years old) critically ill patients in North America and Europe with a CVAD (including CVCs, PICCS, and HD catheters), and investigated any type of locking solution compared to either a saline or heparin control. Outcomes had to include CRBSI/central-line associated bloodstream infection (CLABSI), catheter occlusion, and/or catheter colonization. Only experimental studies were considered. Studies had to have been published in peer-reviewed journals from 2010 onwards. Due to advances in catheter care and management, only papers published in 2010 or later were considered. Only articles published in English were considered for inclusion in the review due to the lack of resources available for translation services.

#### Exclusion criteria

Grey literature including conference abstracts, posters, and case reports were excluded to ensure that only high-quality, peer-reviewed data was used in this systematic review. Published protocols without results were not considered. Table 1 summarizes the research question and parameters considered during literature search and paper selection.

**Table 1:** PICOT Table summarizing research question and parameters of interest.

*Research Question*:	What is the extent and quality of evidence of the effectiveness of various locking solutions in preventing CVAD complications compared to standard of care locking fluids?
***P****opulation*:	Adult (>18 years old) critically ill patients in North America and Europe with a CVAD (including CVCs, PICCS, and hemodialysis catheters)
***I****ntervention*:	CVAD locking solutions: antibiotic, antiseptic, ethanol, citrate, EDTA, taurolidine, heparin
***C****omparator*:	Standard of care locking solutions: saline, heparin, or citrate (depending on the center)
***O****utcomes*:	Any of the following: CLABSI and/or Catheter-Related Bloodstream Infection (CRBSI), catheter occlusion, catheter colonization
***T****ype*:	Randomized, controlled, trials, conducted in North America and Europe, and published in peer-reviewed journals from 2010 onwards.

### Information sources

The literature search was performed by an independent librarian at the Health Sciences Library at Royal Columbian Hospital (New Westminster, Canada). MEDLINE and EMBASE databases were searched on December 30^th^, 2020. An updated search was performed on November 28^th^, 2022, to determine if any new papers had been published since the original search date. Where non-experimental studies (including care guidelines, systematic reviews and meta-analyses, opinion reviews, etc.) found in the electronic search were deemed relevant to the review topic, their reference lists were also reviewed for relevant papers to include in the next stage of abstract review. As this was a review study, no ethics approvals were necessary.

### Search strategy

Search terms were developed in collaboration with the independent librarian. The exact search terms and specifications used can be found in S1 File. The references of any non-experimental papers included in the search result were screened for relevant experimental studies.

### Selection process

Two review authors (MO and JS) independently read the title and abstract of each paper to identify studies selected for full-text review based on the criteria mentioned above. Experimental studies whose interventions, patient populations, or outcomes were unclear based on just the abstract, or papers that did not have an abstract available, were also moved on to full-text review for further assessment. Where interventions, patient populations, or outcomes remained unclear, reviewers also investigated Clinical Trial registration databases, and available supplementary materials to make their final adjudication. Full texts of papers were also screened for mentions of “ICU/Intensive Care Unit/Intensive Care/Critical Care” to ensure desired patient population. Papers with no references to these words were considered to not include patient populations of interest and were excluded from the full-text stage of the review. Finally, eligible papers deemed to fit inclusion and exclusion criteria following the full-text review underwent quality appraisal and final inclusion in the systematic review. Any discrepancies were resolved by a third, independent reviewer (SR).

### Data collection process

Two review authors (MO and JS) independently extracted relevant information from each paper. Where available, Clinical Trial registrations and supplementary materials were also reviewed. Where information was still missing after reviewing those sources, the corresponding authors of each study were contacted with additional questions. Authors were contacted one additional time if there was no response after the first contact attempt.

### Data items

Information about patient population, type of CVAD, number of participants, types of locking fluids compared, and outcomes as stated in the original study were extracted from each paper and used to complete Table 1. Outcomes specific to the study question (CRBSI, catheter colonization, and catheter occlusion) in both percentages per total CVADs and rate per 1000 catheter-days were extracted or calculated from each paper by using the information found in the original publication (in the text, Tables, or Figures), or by contacting study authors for additional information if necessary.

### Study risk of bias assessment

For randomized, controlled trials included in the final set of papers, the Revised Cochrane risk-of bias tool for randomized trials (RoB 2) was utilized [28]. The signaling questions of this tool were first answered independently by MO and JS to appraise the quality and risk of bias of each paper. Any discrepancies in quality appraisal between MO and JS were resolved by a third, independent reviewer (SR). MO and JS both appraised a practice paper [29] as training prior to beginning the quality appraisal process to gain familiarity with using the RoB 2 tool to increase study rigor.

In accordance with the appropriate use of Cochrane risk of bias tools [30], the Risk of Bias In Non-Randomized Studies- of Intervention (ROBINS-I) assessment tool [31] was used for the one quasi-experimental paper included in the final subset of papers for appraisal. Similarly, to the RoB 2, Tables 1 and 2 of the ROBINS-I tool were used to determine the overall risk of bias in the quasi-experimental paper. Quality appraisal was completed in the same manner by MO, JS, and SR as described above.

**Table 2:** Study characteristics.

Author, Year (Total N)	Patient Population	CVAD Types	Locking Intervention, (n)	Control Comparator, (n)	Outcomes	Results
Hermite, 2012 (n = 78)	Adult (>18 years) Medical and Surgical ICU patients with acute renal failure requiring hemodialysis	HD Catheter	Trisodium Citrate, 46.7% (n = 39)	Saline, 0.9% (n = 39)	Catheter lifespan, Catheter malfunction, CRBSI	Median catheter lifespan: Citrate = 12 days, Saline = 6 days, p = 0.0019* Catheter malfunction: Citrate = 26 per 1000 c-d, saline = 127 per 1000 c-d, p < 0.00001* CRBSI: Citrate = 24 per 1000 c-d, Saline = 30 per 1000 c-d, p > 0.05
Schallom, 2012 (n = 341)	Adult (>18 years) medical or surgical/burn/trauma ICU patients	CVC	Saline, 0.9% (n = 165)	Heparin, 10 units per mL (n = 167)	Catheter non-patency, loss of blood return, alteplase use, CRBSI	Catheter non-patency: Saline = 6.3%, Heparin = 3.8%, p = 0.136 Loss of blood return: Saline = 27.8%, Heparin = 22.35, p = 0.091 Alteplase use: Saline = 6.3%, Heparin = 2.8%, p = 0.049 CRBSI: Saline = 3.1 per 1000 c-d, Heparin = 0 per 1000 c-d, p = 0.125
Perez-Granda, 2014 (n = 200)	Adult (>18 years) Cardiac Surgery ICU Patients	CVC	Ethanol, 70% (n = 113)	Heparin (n = 187)	CRBSI, skin colonization, hub colonization, tip colonization, UTI, Bacteremia, VAP, surgical wound injection, clostridium-difficile infection, ICU days of stay, hospital days of stay, hospital mortality	CRBSI: Ethanol = 2.17 per 1000 c-d, Heparin = 5.24 per 1000 c-d, p = 0.33 Skin colonization: Ethanol = 39.52 per 1000 c-d, Heparin = 49.53 per 1000 c-d, p = 0.38 Hub colonization: Ethanol = 4.4 per 1000 c-d, Heparin = 3.82 per 1000 c-d, p = 0.87 Tip colonization: Ethanol = 15.26 per 1000 c-d, Heparin = 8.0 per 1000 c-d, p = 0.19 Urinary Tract Infection: Ethanol = 6.2%, Heparin = 8%, p = 0.61 Bacteremia: Ethanol = 5.5%, Heparin = 7.1%, p = 0.64 Ventilator- associated pneumonia: Ethanol = 1.8%, Heparin = 3.4%, p = 0.45 Surgical wound infection: Ethanol = 2.7%, Heparin = 0%, p = 0.12 Clostridium difficile infection: Ethanol = 1.8%, Heparin = 1.1%, p = 0.63 ICU DOS (days): Ethanol = 5, Heparin = 6, p = 0.75 Hospital DOS (days): Ethanol = 15, Heparin = 16, p = 0.77 Hospital mortality: Ethanol = 6.2%, Heparin = 8%, p = 0.65
Parienti, 2014 (n = 596)	Adult (>18 years) ICU patients requiring renal replacement therapy	HD Catheter	Trisodium Citrate, 46.7% (n = 132)	Heparin or Saline (n = 464)	Catheter tip colonization, catheter dysfunction, CRBSI, duration of catheterization, all-cause ICU mortality	Catheter-tip colonization: Citrate = 14.4%, SOC = 27.6%, p = 0.002* Catheter dysfunction: Citrate = 3.0%, SOC = 9.9%, p = 0.02* CRBSI: Citrate = 0.8%, SOC = 1.3%, p = 0.53 Duration of catheterization: Citrate = 6.1%, SOC = 5.9%, p = 0.84 All-cause ICU mortality: Citrate = 34.1%, SOC = 40.5%, p = 0.18
Souweine, 2015 (n = 1460)	Adult (>18 years) ICU patients requiring renal replacement therapy or plasma exchange	HD Catheter	Ethanol, 60% (n = 730)	Saline, 0.9% (n = 730)	Catheter-related infection, catheter-related clinical sepsis without bloodstream infection, CRBSI, catheter colonization, catheter dysfunction, catheter removed due to dysfunction or obstruction	Catheter-related infection: Ethanol = 3.83 per 1000 c-d, Saline = 2.64 per 1000 c-d, p = 0.17 Catheter-related clinical sepsis without bloodstream infection: Ethanol = 1.8 per 1000 c-d, Saline = 0.47 per 1000 c-d, p = 0.03 CRBSI: Ethanol = 1.99 per 1000 c-d, Saline = 2.17 per 1000 c-d, p = 0.99 Catheter colonization: Ethanol = 15.18 per 1000 c-d, Saline = 12.08 per 1000 c-d, p = 0.57 Catheter dysfunction: Ethanol = 14.6%, Saline = 16%, p = 0.08 Catheter removal due to dysfunction or obstruction: Ethanol = 24.4%, Saline = 22.7%, p = 0.35
Quenot, 2019 (n = 396)	Adult (>18 years) ICU patients requiring hemodialysis	HD Catheter	Trisodium Citrate, 4% (n = 199)	Heparin, 5000 IU/mL (n = 197)	Event-free survival of the first non-tunneled HD catheter, fibrinolysis, catheter dysfunction, catheter-related infection, CRBSI, number of hemorrhagic events requiring transfusion, length of stay in intensive or critical care, length of hospital stay, death rate at 28 days	Event-free survival of the first non-tunneled hemodialysis catheter: Citrate = 7 days, Heparin = 5 days, p = 0.51 Fibrinolysis: Citrate = 2%, Heparin = 0%, p = 1.0 Catheter dysfunction: Citrate = 15%, Heparin = 9.5%, p = not calculated Catheter-related infection: Citrate = 2.47 per 1000 c-d, Heparin = 0.63 per 1000 c-d, p = 0.37 CRBSI: Citrate = 0.68 per 1000 c-d, Heparin = 0 per 1000 c-d, p = 1.0 Number of hemorrhagic events requiring transfusion: Citrate = 11%, Heparin = 14%, p = 0.42 Length of stay in intensive or critical care: Citrate = 6 days, Heparin = 6 days, p = 0.70 Length of hospital stay: Citrate = 15 days, Heparin = 12 days, p = 0.18 Death rate at 28 days: Citrate = 44%, Heparin = 48%, p = 0.49
Pook, 2022 (n = 100)	Adult (>18 years) ICU patients with any CVAD expected to remain in situ for at least 72 hours	Large bore introducer, CVC, PICC, short-term hemodialysis catheters	Chlorhexidine digluconate + Saline, 0.9% (for non-hemodialysis catheters/lumens) or Chlorhexidine digluconate + trisodium citrate, 4% (for hemodialysis catheters/lumens) (n = 50)	Saline, 0.9% (for non-hemodialysis catheters/lumens) or trisodium citrate 4% (for hemodialysis catheters/lumens) (n = 50)	Length of stay in ICU, length of stay in hospital, death at 28 days, bacteremia, proportion of CVAD colonization (# positive blood cultures/ total blood cultures drawn)	Length of stay in ICU: Chlorhexidine = 7 days, SOC = 6 days, p = 0.649 Length of stay in hospital: Chlorhexidine = 18 days, SOC = 13 days, p = 0.485 Death at 28 days: Chlorhexidine = 18%, SOC = 24%, p = 0.649 Bacteremia: Chlorhexidine = 6.1%, SOC = 17.6%, p = 0.065 CVAD colonization proportion: Chlorhexidine = 18.7%, SOC = 29.0%, p = 0.009*

Abbreviations used: per 1000c-d = events per 1000 catheter-days, HIT = heparin-induced thrombocytopenia

* indicates statistically significant result

## Results

### Study selection

The original literature search returned 36 results. Of these papers, the title and abstract of 5 papers were determined to meet inclusion and exclusion criteria and moved to full-text review. Eight non-experimental papers were deemed relevant to the research question; reference lists were reviewed as per the pre-determined protocol described above. In the case where non-experimental papers referenced other non-experimental papers, the reference lists of those papers were also reviewed, and so on, until only a subset of experimental papers was reached. The references of a total of 118 papers were reviewed, and a total of 240 additional papers underwent title and abstract review.

**Fig 1:**
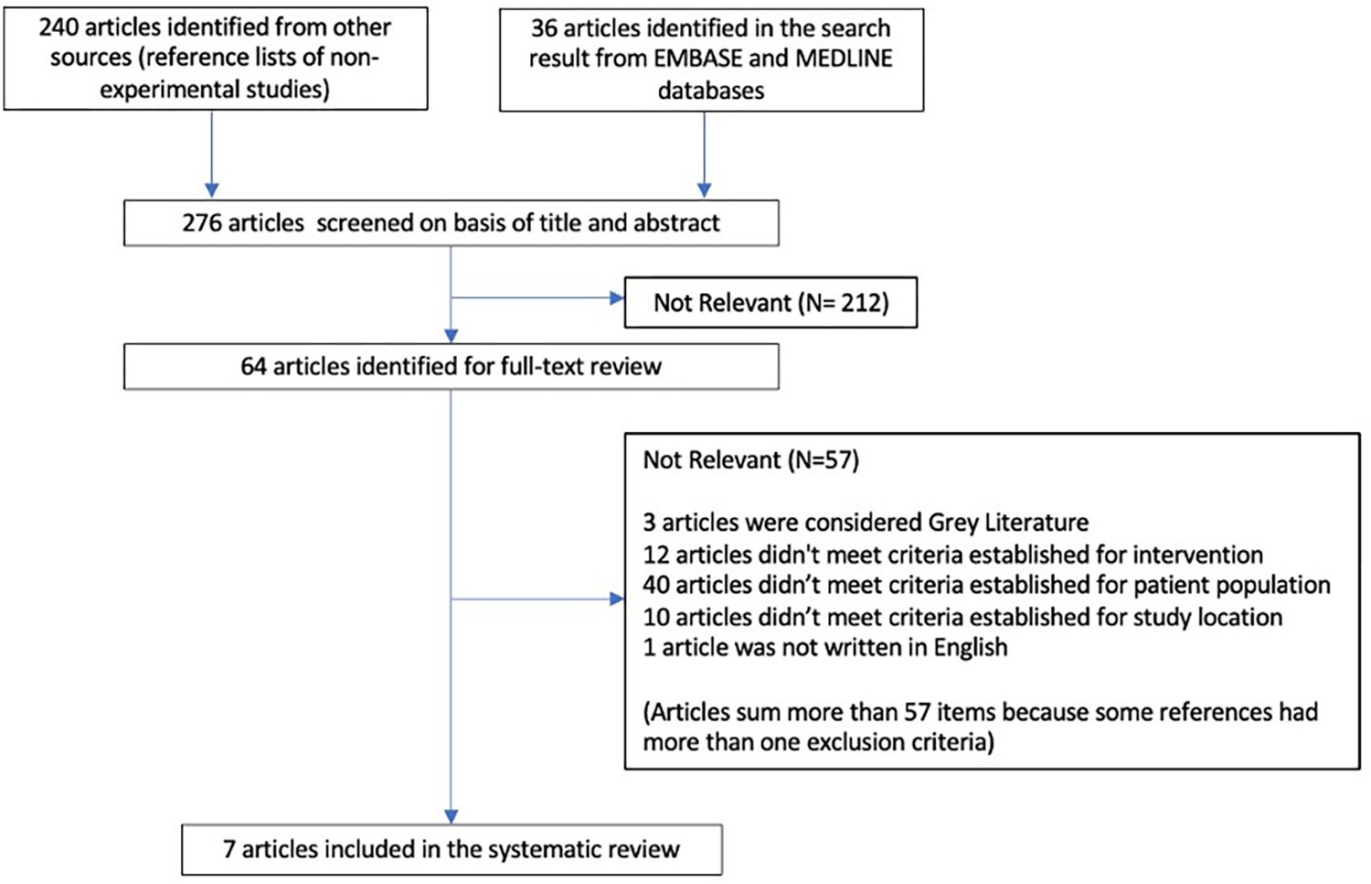
PRISMA flowchart of studies included in the systematic review.

A total of 64 papers underwent full-text review. Of these papers, 6 were deemed eligible to move on to the final stage of quality appraisal. Results of this original search also included one study protocol [32] which was excluded at that time as per exclusion criteria. Published results [33] were included in the final quality appraisal following their publication in 2022. Results of the search repeated on November 28^th^, 2022 returned no further papers to include. See Fig 1.

Most studies were eliminated on the basis of patient population (studies were conducted in long-term or chronic hemodialysis outpatients, patients undergoing chemotherapy, or pediatric/neonatal patients), or were not performed in North America or Europe. Further, other studies were deemed ineligible due to intervention (for example, investigation of different catheter insertion methods, location of catheter insertion, the use of bundles and insertion checklists, catheter decolonization using chlorhexidine bathing, etc.). Of note, few studies appeared to meet most study criteria but were excluded from the review upon further inspection. For example, one study by Jonker et al. [34] was excluded as the intervention was found to be flushing and not locking of CVADs. This retrospective cohort study investigated flushing with heparin versus saline during a nation-wide heparin shortage. Additionally, authors compared rates of alteplase use between groups, which is not included as one of the outcomes investigated in this review.

### Study characteristics

Relevant characteristics of each study as described in the original paper are presented in Table 2 below.

### Risk of bias in studies

**Fig 2:**
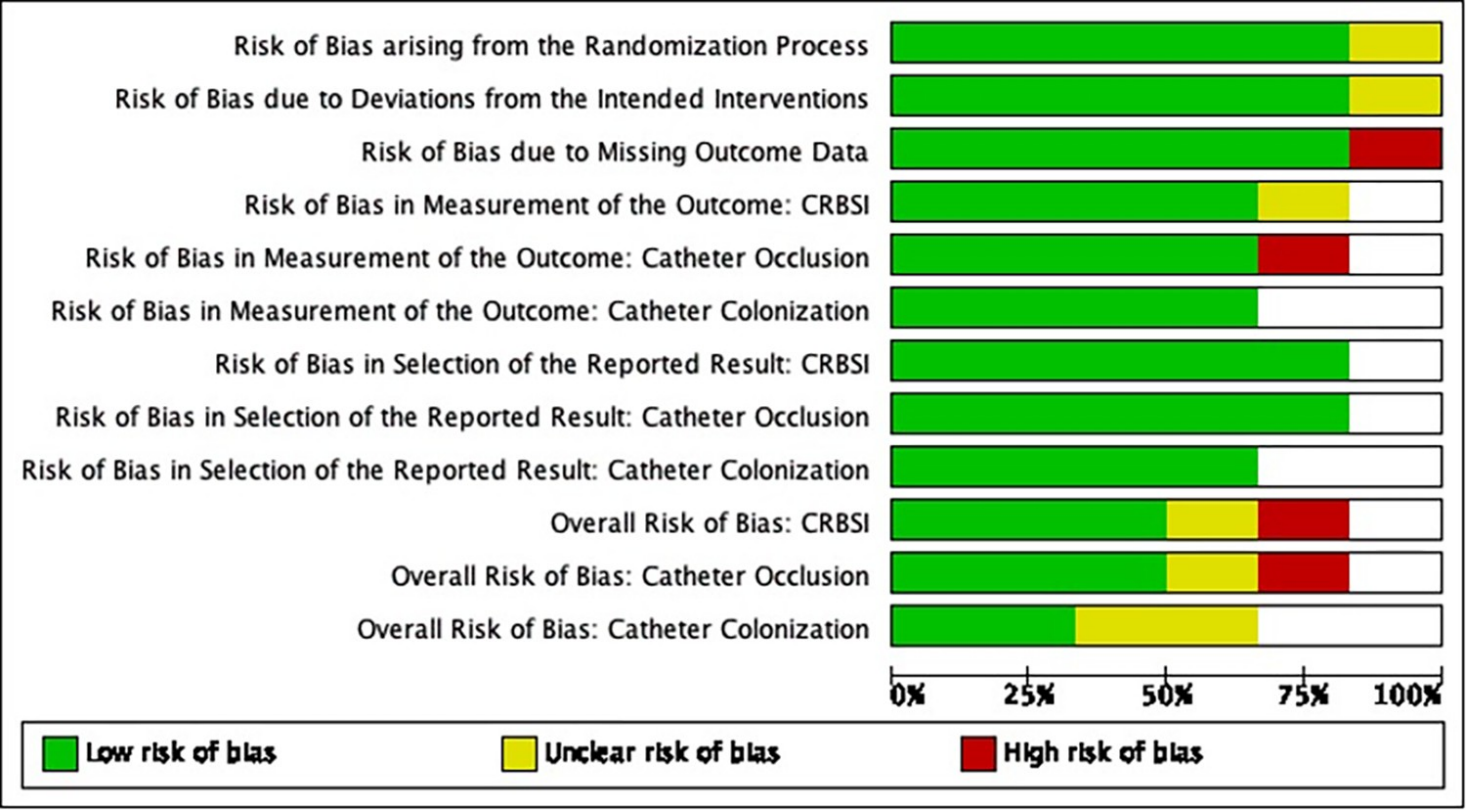
Risk of bias graph of randomized studies. Review authors’ judgements about each risk of bias item presented as percentages across all included studies. Blank spaces are present where a study did not evaluate a specific outcome(s).

**Fig 3:**
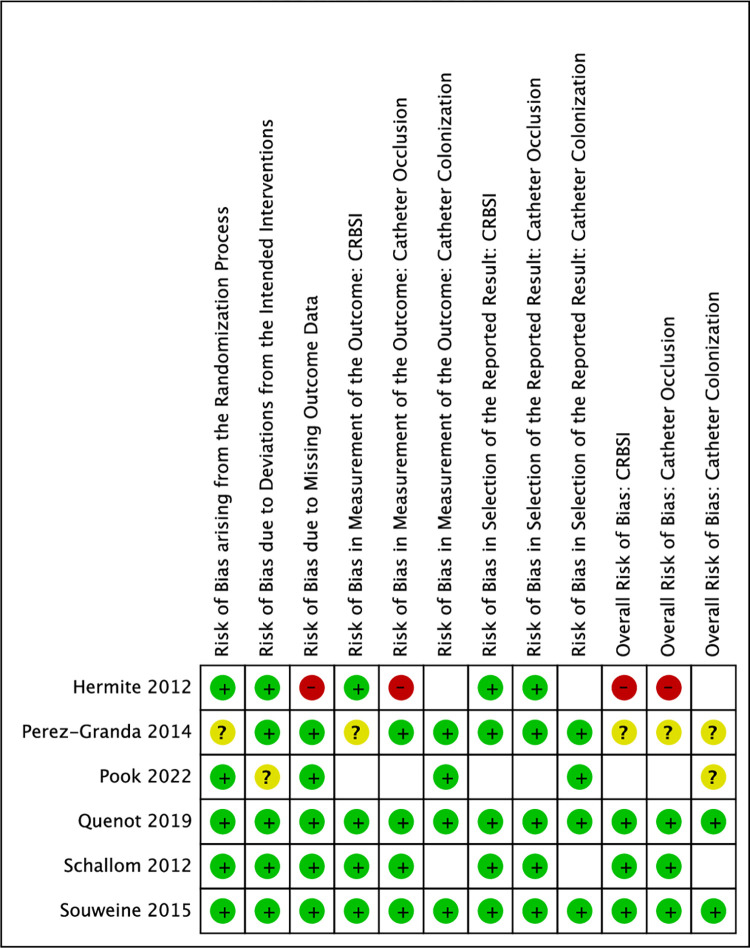
Risk of bias summary of randomized studies. Review authors’ judgements about each risk of bias item for each included study. Blank spaces are present where a study did not evaluate a specific outcome(s).

**Fig 4:**
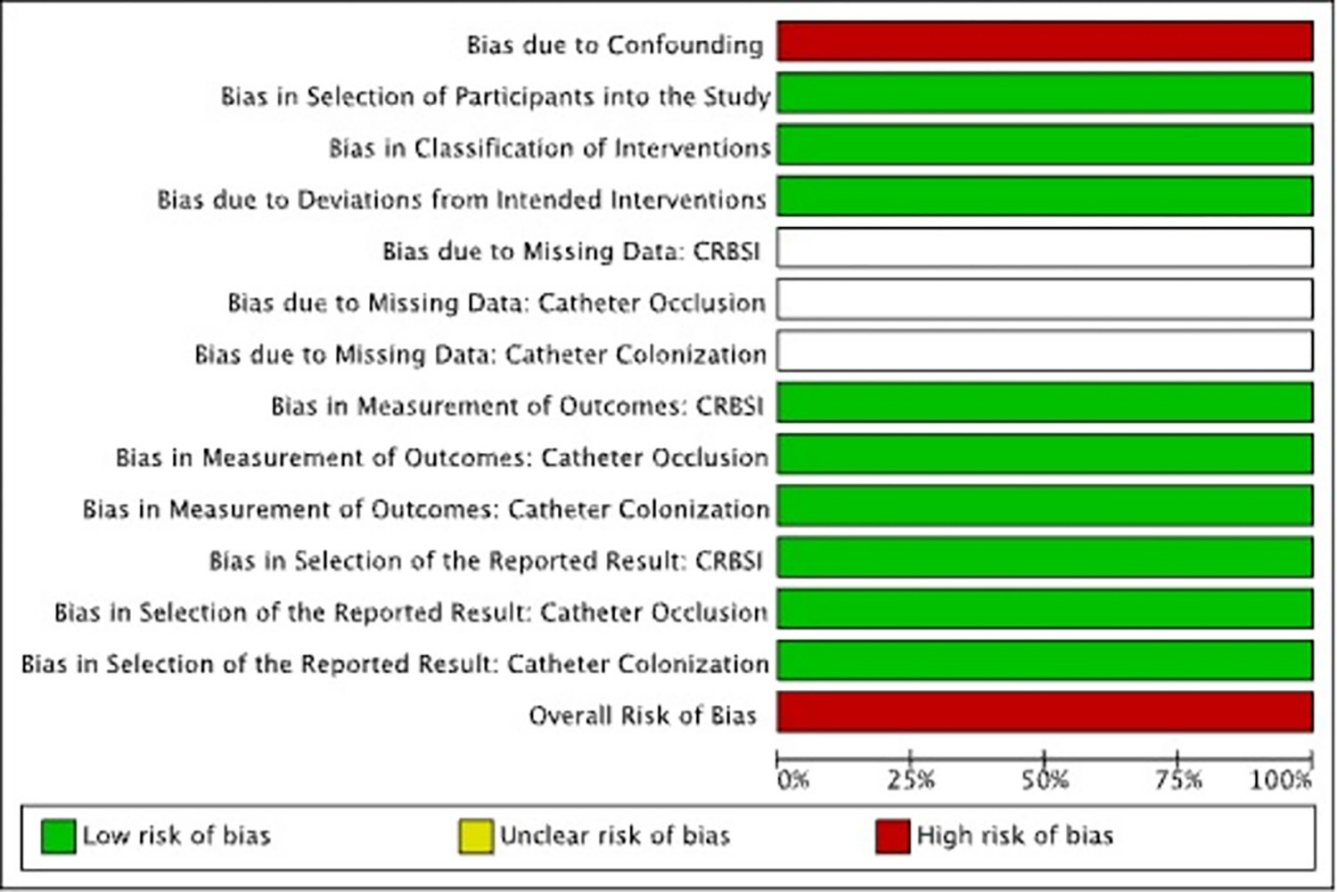
Risk of bias graph, Parienti et al., 2014. Review authors’ judgements about each risk of bias item presented as percentages across all included studies. Blank spaces represent domain categories where there was no information available to adequately assess the domain.

**Fig 5:**
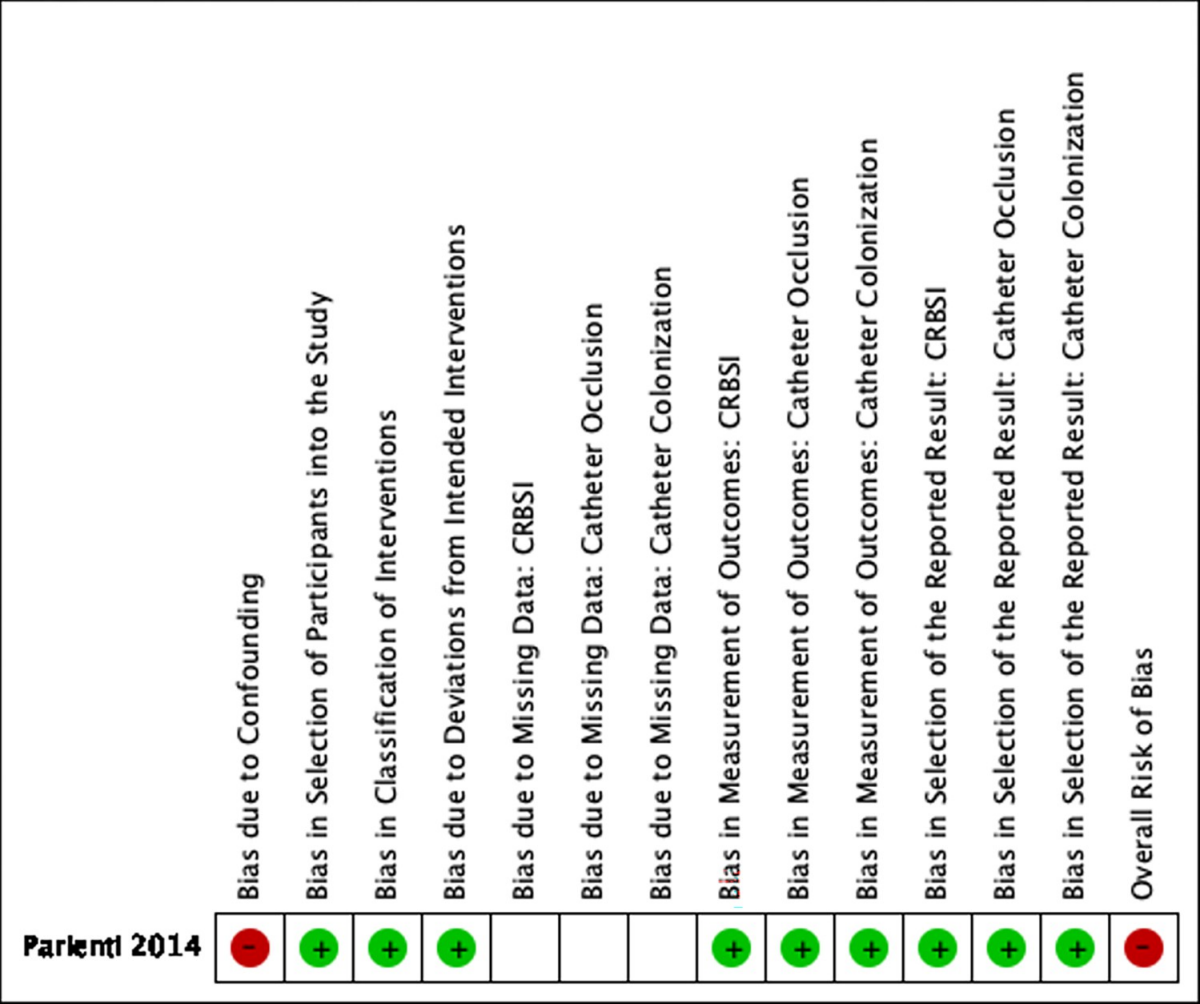
Risk of bias summary, Parienti et al. **2014.** Review authors’ judgements about each risk of bias item for each included study. Blank spaces are present where a study did not evaluate a specific outcome(s).

Results of Quality Appraisals for each paper are summarized in Figs 2–5. Detailed information regarding sources reviewed and justifications for adjudication of risk of bias in each study are presented in S2 File.

### Results of individual studies

Results of individual studies relative to each outcome measure as described in this review are summarized in Tables 3–5. Results are presented in percentages of total CVADs tracked in each study, as well as rates per 1000-catheter days. Additional information was sought from one group of original study authors [35] to separate data from the control group (which comprised of patients who received both heparin and saline locks) into the two separate locking fluid conditions. We have included this data with permission from the corresponding author of that paper.

**Table 3:** Summary of data for CRBSI outcome.

Author, Year	Locking Condition	Total number of CVADs	Catheter-Days	# Cases CRBSI	CRBSI %	CRBSI per 1000 catheter-days
Hermite, 2012	Trisodium citrate, 46.7%	58	685	16	28.345	24
	Saline, 0.9%	77	523	16	20.377	30
Schallom, 2012	Heparin, 10 units/mL	156	1253	0	0	0
	Saline, 0.9%	170	1300	4	2.353	3.077
Perez-Granda, 2014	Ethanol, 70%	179	955	2	1.117	2.094
	Heparin	144	805	4	2.778	4.969
Parienti, 2014	Trisodium citrate, 46.7%	132	909	1	0.758	1.100
	Saline, 0.9%	158	1145	2	1.266	1.747
	Heparin	306	2163	4	1.307	1.849
Souweine, 2015	Ethanol, 60%	1106	6541	13	1.175	1.987
	Saline, 0.9% + 100 U/ml of unfractionated heparin	1066	6496	14	1.313	2.155
Quenot, 2019	Trisodium Citrate, 4%	199	1461	1	0.5	0.680
	Heparin, 5000 IU/mL	197	1590	0	0	0

**Table 4:** Summary of data for CVAD colonization outcome.

Author, Year	Locking Condition	Total number of CVADs	Catheter-Days	# Cases Colonization	Colonization %	Colonization per 1000 catheter-days
Perez-Granda, 2014	Ethanol, 70%	179	955	18	10.056	18.848
	Heparin	144	805	9	6.25	11.180
Parienti, 2014	Trisodium citrate, 46.7%	132	909	19	14.394	20.902
	Saline, 0.9%	158	1145	39	24.684	34.061
	Heparin	306	2163	89	29.085	41.147
Souweine, 2015	Ethanol, 60%	1106	6541	99	8.951	15.135
	Saline, 0.9% + 100 U/ml of unfractionated heparin	1066	6496	78	7.317	12.007
Quenot, 2019	Trisodium Citrate, 4%	199	1461	11	5.528	7.529
	Heparin, 5000 IU/mL	197	1590	9	4.569	5.660

Data from Pook et al. 2022 is not available.

**Table 5:** Summary of data for CVAD occlusion outcome.

Author, Year	Locking Condition	Total number of CVADs	Catheter-Days	# Cases Occlusion	Occlusion %	Occlusion per 1000 catheter-days
Hermite, 2012	Trisodium citrate, 46.7%	58	685	18	30.707	26
	Saline, 0.9%	77	523	66	86.261	127
Schallom, 2012	Heparin, 10 units/mL	156	1253	12	7.692	9.577
	Saline, 0.9%	170	1300	25	14.706	19.231
Perez-Granda, 2014	Ethanol, 70%	179	955	5	2.793	5.236
	Heparin	144	805	4	2.778	4.969
Parienti, 2014	Trisodium citrate, 46.7%	132	909	4	3.030	4.400
	Saline, 0.9%	158	1145	9	5.696	7.860
	Heparin	306	2163	37	12.092	17.106
Souweine, 2015	Ethanol, 60%	1106	6541	270	24.412	41.278
	Saline, 0.9% + 100 U/ml of unfractionated heparin	1066	6496	242	22.702	37.254
Quenot, 2019	Trisodium Citrate, 4%	199	1461	28	14.070	19.165
	Heparin, 5000 IU/mL	197	1590	18	9.137	11.321

CVAD colonization was considered to include results of any positive quantitative tip culture, or if the CVAD was not removed, through-CVAD blood culture.

## Discussion

Despite tremendous clinical efforts and research investigations into the prevention of CVAD complications, they remain a challenge in the adult critical care setting. Utilization of alternative locking fluids with additional prophylactic properties presents an appealing opportunity for intervention, especially when considering the length of time CVADs remain in a locked state in the average ICU patient [36]. In spite of this, only 7 studies [33, 35, 37–41] were identified to fit within the parameters of this review. Further, only five different types of locking fluids have been studied; of which saline, heparin, and citrate are already incorporated into the standard of care. Importantly, the majority of the studies included in this review report no significant differences in rates of complications between locking fluid conditions. As shown in Table 1, only the two studies [35, 37] which investigated citrate at the higher 46.7% concentration show significant differences in median catheter lifespan, CVAD colonization, and occlusion. This is not including the study conducted by Pook et al. [33] whose sample size was determined for feasibility and exploratory purposes only. Unfortunately, the more effective high concentration citrate formulations have been met with safety concerns which led to the FDA discouraging the use of the 46.7% concentration for catheter locking [42]. No adverse events related to citrate were reported in the studies included in this review. None of the studies included in the review show any significant reductions in the development of CRBSI.

Due to the small number of studies included, as well as the narrow range and different combinations of types of locking fluids investigated, it was not possible to perform any type of meta-analysis. Of the two studies that investigated the use of ethanol, locking procedures differed too greatly to facilitate synthesis of data. In the study performed by Souweine et al. [40], the ethanol lock was only instilled for a period of 2 minutes in the intervention group, then aspirated, and subsequently replaced with a standard of care locking fluid (0.9% saline containing 100 U/ml of unfractionated heparin, except for those patients with risk of bleeding where 0.9% saline only was used). The standard of care locking fluid remained in the lumen until subsequent catheter access. Conversely, the study conducted by Perez-Granda et al. [39] included leaving the ethanol instilled for a 2-hour dwell time, and no aspiration prior to re-accessing the line. As such, heterogenous methodology prevented data meta-analysis of the ethanol locking condition.

Similarly, a meta-analysis of data from citrate and chlorhexidine locking conditions across papers could not be completed due to lack of data. Only one study was identified that investigated the use of a chlorhexidine additive to the standard saline lock (for CVCs/PICCs) or 4% citrate lock (for HD catheters). Out of the studies that included citrate as a comparator, the disparity in concentrations used (4% versus 46.7%) was considered to be too large to group all the papers in a single comparison condition. Although the standard of care condition in the study by Pook et al. [33] also included both saline and citrate 4% use (depending on the type of CVAD), authors of the paper did not collect data related to outcomes in each of saline and citrate-locked CVADs separately. Although heparin and saline were used as a comparator for majority of the studies included in the review, only one paper [38] included their direct comparison. As such, meta-analysis was not possible.

It is important to note that outcome definitions used to assess CVAD occlusion were heterogenous between studies, which may undermine the validity of the appraisal. For example, ‘catheter malfunction’ by Hermite et al. [37] was described as “a reduction of 20% or more in blood flow through the catheter, despite attempts to restore patency.” Conversely, Parienti et al. [35] used the term ‘catheter dysfunction’ which was defined by study authors as “inability to attain an adequate blood flow, requiring catheter replacement.” Quenot et al. [41] defined catheter dysfunction as “the inability to achieve and maintain a blood flow of more than 200 mL/min despite changing the patient’s position, inverting the lines and flushing with saline solution.”, while Souweine et al. [40] defined this as “a problem with catheter flow, unfavorable inflow and outflow line pressures requiring catheter mobilization, inversion of lines, and flush through the catheter lumen”. Schallom et al. [38] described ‘lumen non-patency’ as failure to flush the line or aspirate blood following attempts at patient repositioning and cap change.” Meanwhile, Perez-Granda et al. [39] utilized catheter obstruction requiring removal as an outcome definition for CVAD occlusion. Indeed, there is no standardized definition of measuring CVAD occlusion currently utilized across all studies.

Factors that limited the quality of studies identified include not assessing the impact of unaddressed covariates and failure to reach appropriate study power. For example, the effect of time was not adjusted for by Parienti et al. [35] The control cohort was recruited between 2004 and 2007 [43], while the experimental cohort was recruited between 2011 and 2012. Within this period there has been an increased focus on preventing catheter-related complications, for example, with the wide-spread implementation of insertion bundles [44]. Any change of practice to catheter maintenance procedures over time was not considered in the analysis, which was appropriately mentioned as a limitation in the paper. Another limitation amongst included papers was not taking into account important covariates such as the use of antibiotic coated CVADs, or the effect of additional nursing education procedures during analysis [38].

Difficulties with meeting power requirements was a common theme amongst the majority of papers included in this review. Quenot et al., Souweine et al., and Schallom et al. [38, 40, 41] all based power calculations on estimations of baseline rates of complications or catheter lifespan that were not met in control groups of the studies. Additionally, the study by Perez-Granda et al. [39] was terminated at the halfway mark due to adverse events; the sample size in the feasibility study by Pook et al. [33] was not determined based on adequate power to evaluate statistical differences in clinical outcomes. Despite the majority of studies in this review earning a low risk of bias appraisal, major problems with reaching statistical power necessitate that the results of these trials be interpreted with caution. This highlights the importance of performance of pilot observational studies prior to clinical trial planning, as well as the regular surveillance and reporting of baseline CVAD complication rates in the critical care setting and preparation of reviews that summarize this data [9].

Overall, results of the review indicate existing literature on this topic is sparse. Furthermore, it is difficult to interpret with the presence of mixed results, underpowered studies, and inconsistency of methodologies and outcome measures. Not only are ‘alternative’ locking fluids not extensively studied in this patient population, but the strength of evidence for the current standard of care is also limited. Future research directions should include the investigation of a broader variety of alternative locking fluids to the current standard of care. This necessitates standardization of locking methodology and occlusion outcome definitions. It is imperative that future studies are adequately powered, randomized controlled trials in this patient population to facilitate optimal evidence-based care.

## Registration and protocol

This protocol was registered on the International Prospective Register of Systematic Reviews (PROSPERO) database under the record ID: CRD42021239157 on March 27^th^, 2021. One amendment was made on June 18^th^, 2021, which narrowed down the outcomes assessed in the systematic review. This amendment was made following full-text review of papers and determining which outcomes were reported across all papers and considered the most relevant.

## Supporting information

S1 Checklist PRISMA 2020 for abstracts checklist. (DOCX)

S2 Checklist PRISMA 2020 checklist. (DOCX)

S1 File Literature search results. (PDF)

S2 File Quality appraisal justifications for studies included in the systematic review. (PDF)
