# Supplementary material for: Locking solutions for prevention of central venous access device complications in the adult critical care population: A systematic review
Source: PLoS One. 2023 Oct 12;18(10):e0289938. doi: 10.1371/journal.pone.0289938 (PMC10569507; doi:10.1371/journal.pone.0289938)
Supplement: S1 File — (PDF) [file pone.0289938.s003.pdf]

Literature Search Results

Date Completed: 2020-12-30 **Requestor:** Marlena Ornowska

Request: Efficacy of Various Locking Solutions in the ICU- Systematic Review project with Dr. Steve Reynolds

Research Question: What is the effectiveness of various central venous access device (CVAD) locking solutions in preventing complications modifiable by CVAD locking compared to standard of care saline locking fluids (as described by a statistically significant difference between groups)?

Population: Adult (>18 years old) Intensive Care Unit patients in North America and Europe. **Intervention/Exposure:** various CVAD locking solutions- antibiotic, heparin, ethanol, citrate, EDTA, taurolidine. **Comparisons:** Compared to standard of care- saline locking solutions.

Outcomes: Any of the following- CLABSI and/or CRBSI, bloodstream infection, bacterial biofilm formation, occlusion, catheter-related thrombosis (symptomatic and asymptomatic), alteplase use, local infection/phlebitis, premature catheter replacement, length of ICU stay, length of hospital stay, mortality.

Inclusion/Exclusion Criteria:

- Papers meeting the review objectives described above
- Years Considered: must be published within the last 10 years (2010-2020).
- Language considered: English
- Publication status: published in a peer-reviewed journal

Exclude: Case reports, posters, conference abstracts. **Completed By:** Brooke Ballantyne Scott

Search Strategy:

Ovid MEDLINE(R) and In-Process & Other Non-Indexed Citations 1946 to December 29, 2020

1. Central Venous Catheters/
2. Catheterization, Central Venous/
3. Catheter* adj2 central.ti,ab,kw.
4. Central venous access device*.ti,ab,kw.
5. OR/1-4
6. Lock*.ti,ab,kw.
7. Patency.ti,ab,kw.
8. OR/6-7
9. Exp Intensive Care Units/
10. Exp Critical Care/
11. ICU.ti,ab,kw.
12. Intensive care.ti,ab,kw.
13. Critical care.ti,ab,kw.
14. OR/9-13
15. AND/5,8,14
16. Child*.ti,ab,kw.
17. Neonat*.ti,ab,kw.
18. OR/16-17
19. 15 NOT 18

[Limits: no letters, comments or case reports; 2010-present; English language]

Results from Medline only: 23

EMBASE 1996 to 2020 Week 52

1. Central Venous Catheters/
2. Catheterization, Central Venous/
3. Catheter* adj2 central.ti,ab,kw.
4. Central venous access device*.ti,ab,kw.
5. OR/1-4
6. Lock*.ti,ab,kw.
7. Patency.ti,ab,kw.
8. OR/6-7
9. Exp Intensive Care Units/
10. Exp Critical Care/
11. ICU.ti,ab,kw.
12. Intensive care.ti,ab,kw.
13. Critical care.ti,ab,kw.
14. OR/9-13
15. AND/5,8,14
16. Child*.ti,ab,kw.
17. Neonat*.ti,ab,kw.
18. OR/16-17
19. 15 NOT 18

[Limits: no letters, comments, conference abstracts or case reports; 2010-present; English language]

Results from EMBASE only: 28

Total De-duplicated Results: 36

1. G., L.-P., J.E., A.-A., R., G.-A., L., L., R., L., C., C.-O., ... Hinojosa C A. (2020). **Comparison of axillary-atrial and axillary-iliac arteriovenous grafts for hemodialysis access creation**. Journal of Vascular Access, 21(1), 55–59. https://doi.org/http://dx.doi.org/10.1177/1129729819851919
   Objective: The aim of this study was to compare two complex vascular access techniques that utilize the axillary artery as inflow and accesses were created with early cannulation grafts: the axillary-atrial arteriovenous graft versus axillary- iliac arteriovenous graft. Method(s): This is a retrospective study of end-stage renal disease patients with occluded intrathoracic central veins that underwent complex hemodialysis access creation in our institution after failed endovascular recanalization attempts. Patients' demographics, comorbidities, number and types of previous accesses, intraoperative variables, and clinical outcomes were collected and compared. Result(s): Four patients underwent axillary- atrial arteriovenous graft creation with FlixeneTM (AtriumTM, Hudson, NH, USA) grafts, through a midline sternotomy to expose the right atrium; all were successfully implanted and used for hemodialysis within the first 72 h; one patient developed a pseudoaneurysm in the mid-graft portion, requiring surgical repair, and it is currently functional. Eight axillary-iliac arteriovenous grafts were created; all grafts were patent and were utilized within 96 h after placement. At 6 months of follow-up period, five (62 %) of our patients underwent graft thrombectomy, one (12 %) balloon angioplasty at the vein anastomosis secondary to stenosis, and two (25 %) grafts were removed due to infectious complications. Axillary-atrial arteriovenous graft and axillary-iliac arteriovenous graft primary patency rates at 6

months were 75% and 48%, respectively; 6-month secondary patency of the axillary-atrial arteriovenous graft compares favorably against that of axillary-iliac arteriovenous graft (100% vs 75%, respectively). Conclusion(s): Despite the invasiveness, direct atrial outflow procedures remain a valid alternative in carefully selected patients with adequate cardiopulmonary reserve.Copyright © The Author(s) 2019.

2. E., B., B., Y., A., T., E.T., B., Kara E. AO - Yilmaz, B. O. http://orcid. org/000.-0001-7642-4684, Balikci, E., ... Kara, E. (2020). **Surface modification strategies for hemodialysis catheters to prevent catheter-related infections: A review**. Journal of Biomedical Materials Research - Part B Applied Biomaterials. https://doi.org/http://dx.doi.org/10.1002/jbm.b.34701
Insertion of a central venous catheter is one of the most common invasive procedures applied in hemodialysis therapy for end-stage renal disease. The most important complication of a central venous catheter is catheter-related infections that increase hospitalization and duration of intensive care unit stay, cost of treatment, mortality, and morbidity rates. Pathogenic microorganisms, such as, bacteria and fungi, enter the body from the catheter insertion site and the surface of the catheter can become colonized. The exopolysaccharide-based biofilms from bacterial colonies on the surface are the main challenge in the treatment of infections. Catheter lock solutions and systemic antibiotic treatment, which are commonly used in the treatment of hemodialysis catheter-related infections, are insufficient to prevent and terminate the infections and eventually the catheter needs to be replaced. The inadequacy of these approaches in termination and prevention of infection revealed the necessity of coating of hemodialysis catheters with bactericidal and/or antiadhesive agents. Silver compounds and nanoparticles, anticoagulants (e.g., heparin), antibiotics (e.g., gentamicin and chlorhexidine) are some of the agents used for this purpose. The effectiveness of few commercial hemodialysis catheters that were coated with antibacterial agents has been tested in clinical trials against catheter-related infections of pathogenic bacteria, such as Staphylococcus aureus and Staphylococcus epidermidis with promising results. Novel biomedical materials and engineering techniques, such as, surface micro/nano patterning and the conjugation of antimicrobial peptides, enzymes, metallic cations, and hydrophilic polymers (e.g., poly [ethylene glycol]) on the surface, has been suggested recently.Copyright © 2020 Wiley Periodicals LLC

3. H., Y., Q., X., X., W., L., J., J., W., X., M., ... Jiang, A. (2020). **Effects of different catheter replacement methods on catheter service time and complications in hemodialysis patients: A cohort study.** Journal of Vascular Access, 21(4), 497–503. https://doi.org/http://dx.doi.org/10.1177/1129729819891336
Introduction: Central venous catheter insertion for long-term vascular access is not recommended in clinical practice. However, since arteriovenous fistula creation is difficult to perform in some patients, central venous catheter insertion for long-term vascular access is performed. This study aimed to assess the complications and service time of central venous catheters replaced using different methods and to determine the influencing factors of service time. Method(s): Study design: A retrospective observational cohort study. Setting and participants: Patients who underwent tunneled dialysis catheter malfunction (2009-2019) and had to undergo another dialysis catheter insertion were enrolled. Exposures: Ectopic replacement and in situ replacement. Outcome(s): Factors such as age, sex, primary patency rate, secondary patency rate, early complications, and late complications were considered. Analytical approach: This study used a Cox proportional hazards regression model. Result(s): The first and the newly replaced catheter service time were 37.779 +/- 24.563 months and 32.468 +/- 26.638 (25) months in the ectopic group and 37.075 +/- 20.550 months and 26.349 +/- 22.672 months in the in situ group, respectively. In the early service time, the newly replaced catheter resulted in significant bleeding from the tunnel. The first catheter had the least complications, most adequate blood flow, and longest service time. Ectopic catheter replacement and the tip shape of the catheter were the independent factors for catheter service time. Catheter service time increased with age. Conclusion(s): Ectopic catheter replacement can improve the primary

patency rate and auxiliary primary patency rate of catheters. Ectopic catheter replacement may require sufficient surgical skills with digital subtraction angiography, resulting in a better prognosis.Copyright © The Author(s) 2019.

4. Javeri, Y., Jagathkar, G., Dixit, S., Chaudhary, D., Zirpe, K. G., Mehta, Y., ... Jain, R. (2020). **Indian Society of Critical Care Medicine Position Statement for Central Venous Catheterization and Management 2020.** Indian Journal of Critical Care Medicine : Peer-Reviewed, Official Publication of Indian Society of Critical Care Medicine, 24(Suppl 1), S6–S30. https://doi.org/https://dx.doi.org/10.5005/jp-journals-10071-G23183
Background and Purpose: Short-term central venous catheterization (CVC) is one of the commonly used invasive interventions in ICU and other patient-care areas. Practice and management of CVC is not standardized, varies widely, and need appropriate guidance. Purpose of this document is to provide a comprehensive, evidence-based and up-to- date, one document source for practice and management of central venous catheterization. These recommendations are intended to be used by critical care physicians and allied professionals involved in care of patients with central venous lines., Methods: This position statement for central venous catheterization is framed by expert committee members under the aegis of Indian Society of Critical Care Medicine (ISCCM). Experts group exchanged and reviewed the relevant literature. During the final meeting of the experts held at the ISCCM Head Office, a consensus on all the topics was made and the recommendations for final document draft were prepared. The final document was reviewed and accepted by all expert committee members and after a process of peer-review this document is finally accepted as an official ISCCM position paper.Modified grade system was utilized to classify the quality of evidence and the strength of recommendations. The draft document thus formulated was reviewed by all committee members; further comments and suggestions were incorporated after discussion, and a final document was prepared., Results: This document makes recommendations about various aspects of resource preparation, infection control, prevention of mechanical complication and surveillance related to short-term central venous catheterization. This document also provides four appendices for ready reference and use at institutional level., Conclusion: In this document, committee is able to make 54 different recommendations for various aspects of care, out of which 40 are strong and 14 weak recommendations. Among all of them, 42 recommendations are backed by any level of evidence, however due to paucity of data on 12 clinical questions, a consensus was reached by working committee and practice recommendations given on these topics are based on vast clinical experience of the members of this committee, which makes a useful practice point. Committee recognizes the fact that in event of new emerging evidences this document will require update, and that shall be provided in due time., Abbreviations list: ABHR: Alcohol-based hand rub; AICD: Automat...

5. N., Z., M., P., E., M., Fox-Robichaud A.E. AO - Fox-Robichaud, A. E. . O. http://orcid. org/000.-0001-9912-3606, Zamir, N., Pook, M., ... Fox-Robichaud, A. E. (2020). **Chlorhexidine locking device for central line infection prevention in ICU patients: Protocol for an open-label pilot and feasibility randomized controlled trial**. Pilot and Feasibility Studies, 6(1), 26. https://doi.org/http://dx.doi.org/10.1186/s40814-020-0564-9
Background: Critically ill patients in the intensive care unit (ICU) are at risk for central line-associated bloodstream infection (CLABSI) with an incidence up to 6.9 per 1000 catheter days. CLABSI has a significant attributable mortality and increases in-hospital length of stay, readmissions, and costs. Chlorhexidine gluconate (CHG), a broad-spectrum biocide, has been shown to effectively reduce infections including CLABSI; however, few trials have utilized CHG for prevention of central line infections. Our preclinical work has demonstrated a device that diffuses CHG into the intravenous lock solution of central venous catheters and decreases bacterial growth on the catheter lumen. We designed a clinical trial to test the feasibility of using a CHG device in an ICU patient population., Methods: The proposed pilot trial will be a single centre, open-label, two-arm, parallel group feasibility randomized controlled trial (RCT). Participants will

have a central line in situ and will be enrolled within 72 h of admittance to 3 ICUs at a single academic hospital. Exclusion criteria will include suspected infection, chronic indwelling catheters, and CHG allergy. Informed consent will be obtained from eligible participants or their substitute decision maker prior to randomization. Participants will be randomized to receive either usual care or the CHG locking device. Blood cultures will be drawn from all participants every 48 h. The primary objective of this study will be to determine the feasibility of using this protocol to conduct a larger trial. Feasibility will be assessed through the following outcomes: (1) consent rate, (2) recruitment rate, (3) protocol adherence, and (4) comfort level with the device. The secondary objective of this study will be to establish the preliminary efficacy of the device., Discussion: This study will be the first human RCT to investigate a CHG locking device for the prevention of central line infections. Findings from this trial will inform the feasibility of conducting a large RCT and provide preliminary data on the efficacy of a CHG locking device., Trial registration: ClinicalTrials.gov, NCT03309137, registered on October 13, 2017. Copyright © The Author(s). 2020.

6. Hankins, R., Majorant, O. D., Rupp, M. E., Cavalieri, R. J., Fey, P. D., Lyden, E., & Cawcutt, K. A. (2019). **Microbial colonization of intravascular catheter connectors in hospitalized patients.** American Journal of Infection Control, 47(12), 1489–1492.
https://doi.org/https://dx.doi.org/10.1016/j.ajic.2019.05.024
BACKGROUND: Central line-associated bloodstream infections may be due to catheter connector colonization and intraluminal migration of pathogens. We assessed the colonization of the split septum catheter connector system, and subsequently the luer lock catheter connector system., METHODS: This was a prospective, 2 phase, quality improvement study at a tertiary referral center. Each phase of the study was performed over 3 consecutive days in hospitalized patients receiving an active infusion; first with a split septum lever lock connector and second with a luer lock connector and alcohol port protector. The connectors were inoculated onto blood agar plates and incubated. Plates were assessed for microbial growth after 48-72 hours., RESULTS: In phase I, 98 (41.9%) of 234 split septum connectors yielded microbial growth. In phase II, 56 (23.1%) of 243 luer lock connectors yielded microbial growth. In phase II only, there was a significant increased rate of contamination in peripheral catheters compared with all other catheters, and the rate of contamination on the acute care wards was significantly higher when compared with the intensive care units., CONCLUSIONS: Bacterial colonization of the lever lock system was unacceptably high among all catheter types and hospital locations. Transition to luer lock catheter connectors and alcohol port protectors decreased the colonization; however, colonization still remained substantial. Causation of colonization cannot be determined with these results. Copyright © 2019 Association for Professionals in Infection Control and Epidemiology, Inc. Published by Elsevier Inc. All rights reserved.

7. Huang. (2018). **Effect of a patency bundle on central venous catheter complications among hospitalized adult patients: A best practice implementation project**. JBI Database of Systematic Reviews and Implementation Reports, 16(2), 565–586.
https://doi.org/http://dx.doi.org/10.11124/JBISRIR-2016-003340
Objectives: The aim of this project was to improve continuity of care by decreasing central catheter occlusions in adults in two acute care units with high use of central venous catheters. Specific objectives were to introduce a patency bundle and train nurses on thrombolytic administration for timely resolution of catheter occlusions. Introduction: Central venous catheters are a vital component of medical care of acutely ill hospitalized patients. Occluded catheters delay treatment and can increase infection risk. There are bundles of best practices on catheter insertion, dressing and removal. However, a gap exists in nursing guidance for maintaining and restoring catheter patency. Method(s): The project used the Joanna Briggs Institute's tools for promoting practice change. Getting Research into Practice (GRiP) and Practical Application of Clinical Evidence System (PACES) assisted with strategy formation and data analysis. Direct observation and medical record review were used for baseline and follow-up audits. Several

teaching strategies were employed to educate direct care nurses. Result(s): Occlusion rates fluctuated greatly above and below baseline rates. Infection rates did not increase with thrombolytic administration. Nurses' compliance with catheter access and flushing techniques doubled. Timeliness of thrombolytic administration increased to 100% compliance. However, patency documentation did not improve. Conclusion(s): Multi-modal education reached all nurses and contributed to significantly improved compliance with patency best practice. Nurses were empowered to expediently troubleshoot occlusions by using thrombolytics. However, three months of post-implementation data was not enough to demonstrate a continued downward trend in occlusion rates. Thrombolytic administration by nurses on the other adult units and improved patency documentation are in the planning stages.Copyright © 2018 THE JOANNA BRIGGS INSTITUTE.

8. F., C., J., G.-M., J.L., del P., E., B., J.A., C., M., de C., ... Valles, J. (2018). **Executive summary: Diagnosis and Treatment of Catheter-Related Bloodstream Infection: Clinical Guidelines of the Spanish Society of Clinical Microbiology and Infectious Diseases (SEIMC) and the Spanish Society of Intensive Care Medicine and Coronary Units (SEMICYUC).** Enfermedades Infecciosas y Microbiologia Clinica, 36(2), 112–119. https://doi.org/https://dx.doi.org/10.1016/j.eimc.2017.10.019
Catheter-related bloodstream infections (CRBSI) constitute an important cause of hospital-acquired infection associated with morbidity, mortality, and cost. The aim of these guidelines is to provide updated recommendations for the diagnosis and management of CRBSI in adults. Prevention of CRBSI is excluded. Experts in the field were designated by the two participating Societies (Sociedad Espanola de Enfermedades Infecciosas y Microbiologia Clinica and the Sociedad Espanola de Medicina Intensiva, Critica y Unidades Coronarias). Short-term peripheral venous catheters, non-tunneled and long-term central venous catheters, tunneled catheters and hemodialysis catheters are covered by these guidelines. The panel identified 39 key topics that were formulated in accordance with the PICO format. The strength of the recommendations and quality of the evidence were graded in accordance with ESCMID guidelines. Recommendations are made for the diagnosis of CRBSI with and without catheter removal and of tunnel infection. The document establishes the clinical situations in which a conservative diagnosis of CRBSI (diagnosis without catheter removal) is feasible. Recommendations are also made regarding empirical therapy, pathogen-specific treatment (coagulase-negative staphylococci, Sthaphylococcus aureus, Enterococcus spp, Gram-negative bacilli, and Candida spp), antibiotic lock therapy, diagnosis and management of suppurative thrombophlebitis and local complications. Copyright © 2017 Elsevier Espana, S.L.U. and Sociedad Espanola de Enfermedades Infecciosas y Microbiologia Clinica. All rights reserved.

9. Park, H. S., Choi, J., Kim, H. W., Baik, J. H., Park, C. W., Kim, Y. O., ... Jin, D. C. (2018). **Exchange over the guidewire from non-tunneled to tunneled hemodialysis catheters can be performed without patency loss.** The Journal of Vascular Access, 19(3), 252–257. https://doi.org/https://dx.doi.org/10.1177/1129729817747541 PURPOSE: The exchange from a non-tunneled hemodialysis catheter to a tunneled one over a guidewire using a previous venotomy has been reported to be safe. However, some concerns that it may increase infection risk prevent its clinical application. This approach seems particularly useful for acute kidney injury patients requiring initial renal replacement therapy, in whom we frequently worry about the choice of non-tunneled versus tunneled catheters., MATERIALS AND METHODS: From March 2012 to February 2016, 88 cases to receive the over-the-guidewire exchange method from a non-tunneled to a tunneled catheter and 521 cases to receive de novo tunneled catheter placement from the hemodialysis vascular access cohort were compared retrospectively., RESULTS: The immediate complication, later catheter dysfunction requiring replacement, and infection rates were comparable between the two groups. Newly placed tunneled catheter survival in the over-the-guidewire exchange group was comparable with survival in the de novo tunneled catheter group ($p = 0.24$). In addition, when we compared the same two methods

among only intensive care unit patients; they remained similar (p = 0.19)., CONCLUSION: An exchange with the over-the-guidewire method from a non-tunneled to a tunneled catheter was comparable to a de novo catheter placement technique. Therefore, this method should be viewed more favorably and should especially be considered for acute kidney injury patients.

10. Girardot, T., Monard, C., Rimmele, T., T., G., C., M., & Rimmele T. (2018). **Dialysis catheters in the ICU: selection, insertion and maintenance**. Current Opinion in Critical Care, 24(6), 469–475. https://doi.org/http://dx.doi.org/10.1097/MCC.0000000000000543
PURPOSE OF REVIEW: Choosing the best catheter for renal replacement therapy (RRT) is not an easy task. Beyond catheter length, many of its properties can influence effectiveness of the RRT session. Maintenance between sessions, particularly the locking solution, also impacts catheter lifespan and infection rates. RECENT FINDINGS: Many innovations in dialysis catheters have been proposed by the industry over the past decade, including the material used, the shape of the lumens and the position of the inflow and outflow holes. Impregnated catheters have also been developed to prevent catheter-related infections. Many locking solutions are available, either for maintaining catheter patency or for preventing infections. SUMMARY: Although studies conducted in the specific context of the ICU are still scarce, some conclusions can be drawn. Catheter length must be adapted to the insertion site to reach an area of high blood flow. Kidney-shape lumens appear to be less thrombogenic and seem to prevent catheter dysfunction. Catheter tip and lumen holes also affect catheter function. For catheter locking, 4% citrate appears nowadays as one of the best options, but taurolidine-based solutions are also interesting.

11. Chaves, F., Garnacho-Montero, J., Del Pozo, J. L., Bouza, E., Capdevila, J. A., de Cueto, M., ... Valles J. (2018). **Diagnosis and treatment of catheter-related bloodstream infection: Clinical guidelines of the Spanish Society of Infectious Diseases and Clinical Microbiology and (SEIMC) and the Spanish Society of Spanish Society of Intensive and Critical Care Medicine and Coronary Units (SEMICYUC)**. Medicina Intensiva, 42(1), 5–36. https://doi.org/https://dx.doi.org/10.1016/j.medin.2017.09.012
Catheter-related bloodstream infections (CRBSI) constitute an important cause of hospital-acquired infection associated with morbidity, mortality, and cost. The aim of these guidelines is to provide updated recommendations for the diagnosis and management of CRBSI in adults. Prevention of CRBSI is excluded. Experts in the field were designated by the two participating Societies (the Spanish Society of Infectious Diseases and Clinical Microbiology and [SEIMC] and the Spanish Society of Spanish Society of Intensive and Critical Care Medicine and Coronary Units [SEMICYUC]). Short-term peripheral venous catheters, non-tunneled and long-term central venous catheters, tunneled catheters and hemodialysis catheters are covered by these guidelines. The panel identified 39 key topics that were formulated in accordance with the PICO format. The strength of the recommendations and quality of the evidence were graded in accordance with ESCMID guidelines. Recommendations are made for the diagnosis of CRBSI with and without catheter removal and of tunnel infection. The document establishes the clinical situations in which a conservative diagnosis of CRBSI (diagnosis without catheter removal) is feasible. Recommendations are also made regarding empirical therapy, pathogen-specific treatment (coagulase-negative staphylococci, Staphylococcus aureus, Enterococcus spp., Gram-negative bacilli, and Candida spp.), antibiotic lock therapy, diagnosis and management of suppurative thrombophlebitis and local complications. Copyright © 2017 Elsevier Espana, S.L.U. y SEMICYUC. All rights reserved.

12. Kowalewska, P. M., Petrik, S. M., Di Fiore, A. E., & Fox-Robichaud, A. E. (2018). **Antimicrobial Efficacy of a New Chlorhexidine-based Device Against Staphylococcus aureus Colonization of Venous Catheters.** Journal of Infusion Nursing : The Official Publication of the Infusion Nurses Society, 41(2), 103–112.
https://doi.org/https://dx.doi.org/10.1097/NAN.0000000000000267

13. Vascular catheters are a major cause of nosocomial bloodstream infections. ChloraLock (ATTWILL Medical Solutions, Inc, West Jordan, UT, and ICU Medical, Inc, San Clemente, CA) is a novel antimicrobial device containing chlorhexidine digluconate (CHG) that is fitted onto a syringe and infuses CHG into the catheter lumen during locking. The objective of this study was to evaluate the antimicrobial efficacy of ChloraLock with in vitro tests and its ability to reduce Staphylococcus aureus contamination of catheters in the external jugular veins of Yorkshire swine. ChloraLock significantly reduced the bacterial load in the in vitro tests by up to 6 log10 colony-forming units (CFU) and by 3 to 4 log10 CFU/lumen in vivo in a swine model with 0.9% NaCl catheter locks.

14. M.Z.A., M., K.R., C., D.K., M., A.K., G., & Young P J. (2017). **Preventing Retained Central Venous Catheter Guidewires: A Randomized Controlled Simulation Study Using a Human Factors Approach**. Anesthesiology, 127(4), 658–665. https://doi.org/http://dx.doi.org/10.1097/ALN.0000000000001797
Background: Retained central venous catheter guidewires are never events. Currently, preventative techniques rely on clinicians remembering to remove the guidewire. However, solutions solely relying upon humans to prevent error inevitably fail. A novel locked procedure pack was designed to contain the equipment required for completing the procedure after the guidewire should have been removed: suture, suture holder, and antimicrobial dressings. The guidewire is used as a key to unlock the pack and to access the contents; thereby, the clinician must remove the guidewire from the patient to complete the procedure. Method(s): A randomized controlled forced-error simulation study replicated catheter insertion. We created a retained guidewire event and then determined whether clinicians would discover it, comparing standard practice against the locked pack. Result(s): Guidewires were retrieved from 2/10 (20%) standard versus 10/10 (100%) locked pack, n = 20, P < 0.001. In the locked pack group, participants attempted to complete the procedure; however, when unable to access the contents, this prompted a search for the key (guidewire). Participants discovered the guidewire within the catheter lumen, recovered it, utilized it to unlock the pack, and finish the procedure. A structured questionnaire reported that the locked pack also improved subjective safety of central venous catheter insertion and allowed easy disposal of the sharps and guidewire (10/10). Conclusion(s): The locked pack is an engineered solution designed to prevent retained guidewires. Utilizing forced-error simulation testing, we have determined that the locked pack is an effective preventative device and is acceptable to clinicians for improving patient safety.© Copyright 2017, the American Society of Anesthesiologists, Inc. Wolters Kluwer Health, Inc. All Rights Reserved.

15. Hrdy, O., Strazevska, E., Suk, P., Vach, R., Karlik, R., Jarkovsky, J., ... Gal R. (2017). **Central venous catheter- related thrombosis in intensive care patients - incidence and risk factors: A prospective observational study**. Biomedical Papers of the Medical Faculty of the University Palacky, Olomouc, Czechoslovakia, 161(4), 369–373. https://doi.org/http://dx.doi.org/10.5507/bp.2017.034
BACKGROUND: One of the complications associated with central venous catheter (CVC) placement is catheter related deep vein thrombosis (CR-DVT). However a literature search revealed little evidence of this recognised complication. The primary aim of this study was to establish the incidence rate and risk factors for the development of CR-DVT in our critically ill adult patients., METHODS: All critically ill adult patients admitted to the medical-surgical ICU with CVC inserted were included in this observational prospective study. After catheter removal we performed duplex ultrasound examination to assess the patency of the vein and establish if CR-DVT was present., RESULTS: A total number of 308 catheters met the inclusion criteria of which 198 were included in the statistical analysis. The CVC was inserted into a subclavian vein (SCV) in 139 (70%) cases and in an internal jugular vein (IJV) in 59 (30%) cases. The 28-day mortality rate was 14.1%. We found CR-DVT during duplex ultrasound examination in 47 (26%) of all

cases. 33 (70%) of the CR- DVT were diagnosed in the IJV and 14 (30%) in the SCV. The risk factors for the development of CR-DVT we identified included cannulation of the IJV and the use of treatment dose of LMWH. The effect of CR-DVT on 28-day mortality was not statistically significant., CONCLUSION: The risk factors for CR-DVT we identified were IJV as a site for CVC cannulation and the use of therapeutic anticoagulation prior to cannulation. Our recommendation would be preferential cannulation of a subclavian vein as opposed to an internal jugular vein in order to reduce the risk of CR-DVT.

16. J.-A.S., W., P., M., S.D., S., C., D., S., D., N., S., & Tennankore K. (2016). **Comparison of intensive versus standard hemodialysis central venous catheter dysfunction protocol using rt-PA: A quality assurance initiative**. Journal of Vascular Access, 17(2), 143–150. https://doi.org/http://dx.doi.org/10.5301/jva.5000501
Purpose: Catheter locking solutions such as recombinant tissue plasminogen activator (rt-PA) are used to treat and prevent clotting of hemodialysis (HD) catheters during HD treatments and the interdialytic period. However, evidence to guide the use of rt-PA for catheter dysfunction is limited. Method(s): We evaluated outcomes using two catheter dysfunction protocols in a cohort of consecutive prevalent dialysis patients (Jan 2013 to Sep 2014) undergoing HD with a tunneled catheter. In the intensive protocol, rt-PA was administered to all catheters based on blood flow and/or line reversal. In the standard protocol, rt-PA administration was based only on blood flow. The primary outcome was the rate of rt-PA use for catheter malfunction (rt-PA treatment days/1000 total line days; [TLD]). Secondary outcomes included the cost of rt-PA/TLD and the rate of catheter-related bacteremia. Result(s): There were 26 and 35 patients managed by the intensive and standard protocols, respectively. The rate of rt-PA use was 52/1000 TLD (intensive) versus 39/1000 TLD (standard) (rate ratio 1.30, 95% CI 1.12-1.52 CI, $p<0.001$). The rate of bacteremia was 0.43 and 0.22/1000 TLD for the intensive and standard protocols, respectively ($p = 0.491$). The cost of rt-PA was CDN $5.58 and CDN $6.15 per TLD for the intensive protocol and standard protocol groups ($p<0.001$). Conclusion(s): Managing catheter dysfunction based on line reversal and blood flow as opposed to only blood flow was associated with a higher rate of rt-PA use, but at a reduced overall cost.Copyright © 2016 Wichtig Publishing.

17. Lai, N. A. M., Chaiyakunapruk, N., Lai, N. A. M., O'Riordan, E., Pau, W. S. C., Saint, S., ... Saint S. (2016). **Catheter impregnation, coating or bonding for reducing central venous catheter-related infections in adults.** The Cochrane Database of Systematic Reviews, 2016(3), CD007878. https://doi.org/http://dx.doi.org/10.1002/14651858.CD007878.pub3
BACKGROUND: The central venous catheter (CVC) is essential in managing acutely ill patients in hospitals. Bloodstream infection is a major complication in patients with a CVC. Several infection control measures have been developed to reduce bloodstream infections, one of which is impregnation of CVCs with various forms of antimicrobials (either with an antiseptic or with antibiotics). This review was originally published in June 2013 and updated in 2016., OBJECTIVES: Our main objective was to assess the effectiveness of antimicrobial impregnation, coating or bonding on CVCs in reducing clinically-diagnosed sepsis, catheter-related blood stream infection (CRBSI), all-cause mortality, catheter colonization and other catheter-related infections in adult participants who required central venous catheterization, along with their safety and cost effectiveness where data were available. We undertook the following comparisons: 1) catheters with antimicrobial modifications in the form of antimicrobial impregnation, coating or bonding, against catheters without antimicrobial modifications and 2) catheters with one type of antimicrobial impregnation against catheters with another type of antimicrobial impregnation. We planned to analyse the comparison of catheters with any type of antimicrobial impregnation against catheters with other antimicrobial modifications, e.g. antiseptic dressings, hubs, tunnelling, needleless connectors or antiseptic lock solutions, but did not find any relevant studies. Additionally, we planned to conduct subgroup analyses based on the length of catheter use, settings or levels of care (e.g. intensive care unit, standard ward and oncology unit), baseline risks, definition of sepsis, presence or absence of co-interventions and cost-

effectiveness in different currencies., SEARCH METHODS: We used the standard search strategy of the Cochrane Anaesthesia, Critical and Emergency Care Review Group (ACE). In the updated review, we searched the Cochrane Central Register of Controlled Trials (CENTRAL; 2015, Issue 3), MEDLINE (OVID SP; 1950 to March 2015), EMBASE (1980 to March 2015), CINAHL (1982 to March 2015), and other Internet resources using a combination of keywords and MeSH headings. The original search was run in March 2012., SELECTION CRITERIA: We included randomized controlled trials (RCTs) that assessed any type of impregnated catheter against either non-impregnated catheters or catheters with another type of impregnation in adult pati...

18. I., R., A.-M., C., R., Z., M., J., Z., A. H., Y., J., ... J., R. (2016). **Successful salvage of central venous catheters in patients with catheter-related or central line-associated bloodstream infections by using a catheter lock solution consisting of minocycline, EDTA, and 25% ethanol**. Antimicrobial Agents and Chemotherapy, 60(6), 3426–3432. https://doi.org/http://dx.doi.org/10.1128/AAC.02565-15
In cancer patients with long-term central venous catheters (CVC), removal and reinsertion of a new CVC at a different site might be difficult because of the unavailability of accessible vascular sites. In vitro and animal studies showed that a minocycline- EDTA-ethanol (M-EDTA-EtOH) lock solution may eradicate microbial organisms in biofilms, hence enabling the treatment of central line-associated bloodstream infections (CLABSI) while retaining the catheter in situ. Between April 2013 and July 2014, we enrolled 30 patients with CLABSI in a prospective study and compared them to a historical group of 60 patients with CLABSI who had their CVC removed and a new CVC inserted. Each catheter lumen was locked with an M-EDTA-EtOH solution for 2 h administered once daily, for a total of 7 doses. Patients who received locks had clinical characteristics that were comparable to those of the control group. The times to fever resolution and microbiological eradication were similar in the two groups. Patients with the lock intervention received a shorter duration of systemic antibiotic therapy than that of the control patients (median, 11 days versus 16 days, respectively; $P < 0.0001$), and they were able to retain their CVCs for a median of 74 days after the onset of bacteremia. The M-EDTA- EtOH lock was associated with a significantly decreased rate of mechanical and infectious complications compared to that of the CVC removal/reinsertion group, who received a longer duration of systemic antimicrobial therapy. (This study has been registered at ClinicalTrials.gov under registration no. NCT01539343).Copyright © 2016, American Society for Microbiology. All Rights Reserved.

19. M., K., H., K., S.-K., H., Y., H., H., P., J.Y., C., ... Cho Y.-P. (2015). **Surgical treatment of central venous catheter related septic deep venous thrombosis**. European Journal of Vascular and Endovascular Surgery, 49(6), 670–675. https://doi.org/http://dx.doi.org/10.1016/j.ejvs.2015.01.023
Objective/Background The aim of this study was to evaluate the clinical features and outcomes of catheter related central venous thrombosis and whether a surgical approach can be an effective treatment modality in selected cases that are refractory to conservative management. Methods This was a retrospective review of the 46 consecutive patients who were suspected of having central venous catheter related infected deep venous thrombosis and who met the eligibility criteria. Results Conservative management achieved clinical improvement in 26 (56.5%) patients and failed in 20 (43.5%), of whom surgical thrombectomy was performed in 13. The remaining seven patients died before surgery could be performed or their clinical condition was too poor. Apart from one case of wound hematoma (7.7%), post- operative complications that related to the surgical procedure were not observed. Patency of the involved vein was re- established in 12 of the 13 (92.3%) surgically treated patients, and clinical improvement was achieved in 11 (84.6%). In particular, the five patients whose blood cultures revealed Candida species exhibited prompt defervescence after surgical thrombectomy. Conclusion Although conservative management is the first therapy of choice in patients with central venous catheter related infected thrombosis, surgical treatment that removes the septic material can be regarded as a

last resort in critically ill patients with septic thrombophlebitis that is refractory to conservative management.Copyright © 2015 European Society for Vascular Surgery.

20. S.C., S., & Liu K D. (2015). **Finding the key to dialysis catheter lock**. American Journal of Respiratory and Critical Care Medicine, 191(9), 972–974. https://doi.org/http://dx.doi.org/10.1164/rccm.201502-0330ED

21. Y., Z., Y., F., J., Y., Q., A., J., L., N., P., ... Wang Z. (2015). **Synergy of ambroxol with vancomycin in elimination of catheter-related Staphylococcus epidermidis biofilm in vitro and in vivo**. Journal of Infection and Chemotherapy, 21(11), 808–815. https://doi.org/http://dx.doi.org/10.1016/j.jiac.2015.08.017
Central venous catheters are widely used in neonatal intensive care units (NICUs) nowadays. The commonest cause of catheter-related bloodstream infections (CRBSIs) is coagulase-negative staphylococci (CoNS). Ambroxol, an active metabolite of bromhexine, exhibits antimicrobial activity against strains producing biofilm and enhances the bactericidal effect of some antibiotic by breaking the structure of biofilm. In this study, we aimed to determine the effect of ambroxol with vancomycin on the biofilm of Staphylococcus epidermidis (S. epidermidis) in vitro and in vivo. In the in vitro study, the biofilm of S. epidermidis was assessed by XTT reduction assay and analysed by confocal laser scanning microscopy (CLSM). In the in vivo study, a rabbit model of CRBSIs was created by intravenous intubation with a tube covered with S. epidermidis biofilm. The rabbits received one of the following four treatments by means of antibiotic lock therapy: normal heparin, ambroxol, vancomycin, or vancomycin plus ambroxol each for 3 days. The microstructure of the biofilm was assessed by scanning electron microscopy (SEM). The number of bacterial colonies in the organs (liver, heart, and kidney) and on the intravenous tubes was measured on agar plates. Pathological changes in the organs (liver, heart, and kidney) were observed with Hematoxylin-Eosin staining. The ambroxol exhibits significant efficacy to potentiate the bactericidal effect of vancomycin on S. epidermidis biofilm both in vitro and in vivo. The antibiotic lock therapy using a combination of ambroxol and vancomycin reveals a high ability to eradicate S. epidermidis biofilms in vivo. These results provide the basis of a useful anti-infection strategy for the treatment of CRBSIs.Copyright © 2015 Japanese Society of Chemotherapy and The Japanese Association for Infectious Diseases.

22. J.E., T., S.J., M., & Tan K. (2015). **Prevention of central venous catheter-related infection in the neonatal unit: A literature review**. Journal of Maternal-Fetal and Neonatal Medicine, 28(10), 1224–1230. https://doi.org/http://dx.doi.org/10.3109/14767058.2014.949663
Central venous catheter infections are the leading cause of healthcare-associated bloodstream infections and contribute significantly to mortality and morbidity in neonatal intensive care units. Moreover, infection poses significant economic consequence which increased hospital costs and increased length of hospital stay. Prevention strategies are detailed in guidelines published by the Centers for Disease Control and Prevention (CDC) in the United States; nevertheless, recent surveys in neonatal units in the United States, and Australia and New Zealand demonstrate these are not always followed. This review discusses the numerous evidence-based strategies to prevent catheter infections including hand hygiene, maximal sterile barriers during insertion, skin disinfection, selection of insertion site, dressings, aseptic non- touch technique, disinfection of catheter hubs/ports, administration set management, prompt removal of catheter, antibiotic locks, systemic antibiotic prophylaxis and chlorhexidine bathing. Furthermore, it will describe different strategies that can be implemented into clinical practice to reduce infection rates. These include the use of care bundles including checklists, education and the use of CVC teams.Copyright © 2014 Informa UK Ltd.

23. D., H., & Lawrence S. (2015). **The Influence of a Novel Needleless Valve on Central Venous Catheter Occlusions in Pediatric Patients**. JAVA - Journal of the Association for Vascular Access, 20(4), 214. https://doi.org/http://dx.doi.org/10.1016/j.java.2015.07.003 Background Although it is common for central line catheters to develop a thrombotic occlusion, pediatric patients are at especially high risk of occlusion due to smaller vessels, smaller-gauge catheters, and slower rates of infusion. Mitigating catheter occlusions is costly, requiring tissue plasminogen activator, supplies, and nursing time. Our facility tested a novel neutral displacement needle-free valve designed to reduce occlusion. Methodology The organization determined a baseline occlusion rate for Hickman/Broviac catheters, in our 38-bed inpatient hematology/oncology department and our outpatient hematology/oncology clinic from August 2010 through October 2010. In 2011, a premarket test of the Neutron device (ICU Medical, San Clemente, CA) was conducted on the units. Based on the positive trial results, it was decided to implement the device housewide in December 2012. Results A comparison of baseline central line complete occlusion rates from August to October 2010 with Neutron trial data from July to October 2011. This pilot project demonstrated a 74.3% reduction (from a rate of 3.82 to a rate of 0.98) in all hematology/oncology department Hickman and Broviac complete catheter occlusions. Subsequently, comparing 5 months of housewide occlusion data from June through October 2012 to 2013, complete occlusions fell by 32.1% (from a rate of 1.56 to a rate of 1.06). Conclusions The use of the Neutron needle-free catheter patency device was associated with a reduction in complete occlusions. The corresponding reduction in treatment delays, nursing time spent managing occluded catheters, and fewer needlesticks to patients likely translates to financial benefit for the organization and improved patient and family satisfaction.Copyright © 2015 Association for Vascular Access.

24. Souweine, B., Lautrette, A., Gruson, D., Canet, E., Klouche, K., Argaud, L., ... Timsit, J.-F. (2015). **Ethanol lock and risk of hemodialysis catheter infection in critically ill patients. A randomized controlled trial.** American Journal of Respiratory and Critical Care Medicine, 191(9), 1024–1032. https://doi.org/https://dx.doi.org/10.1164/rccm.201408-1431OC RATIONALE: Ethanol rapidly eradicated experimental biofilm. Clinical studies of ethanol lock to prevent catheter-related infections (CRIs) suggest preventive efficacy. No such studies have been done in intensive care units (ICU)., OBJECTIVES: To determine whether ethanol lock decreases the risk of major CRI in patients with short-term dialysis catheters (DCs)., METHODS: A randomized, double-blind, placebo-controlled trial was performed in 16 ICUs in seven university hospitals and one general hospital in France between June 2009 and December 2011. Adults with insertion of a nontunneled, nonantimicrobial-impregnated double-lumen DC for an expected duration greater than 48 hours, to perform renal-replacement therapy or plasma exchange, were randomly allocated (1:1) to receive a 2-minute catheter lock with either 60% wt/wt ethanol solution (ethanol group) or 0.9% saline solution (control group) at the end of DC insertion and after each renal-replacement therapy or plasma exchange session. The main outcome was major CRI defined as either catheter-related clinical sepsis without bloodstream infection or catheter-related bloodstream infection during the ICU stay., MEASUREMENTS AND MAIN RESULTS: The intent-to-treat analysis included 1,460 patients (2,172 catheters, 12,944 catheter-days, and 8,442 study locks). Median DC duration was 4 days (interquartile range, 2-8) and was similar in both groups. Major CRI incidence did not differ between the ethanol and control groups (3.83 vs. 2.64 per 1,000 catheter-days, respectively; hazard ratio, 1.55; 95% confidence interval, 0.83-2.87; P = 0.17). No significant differences occurred for catheter colonization (P = 0.57) or catheter-related bloodstream infection (P = 0.99)., CONCLUSIONS: A 2-minute ethanol lock does not decrease the frequency of infection of DCs in ICU patients. Clinical trial registered with www.clinicaltrials.gov (NCT 00875069).

25. Ibeas-Lopez, J. (2015). **New technology: heparin and antimicrobial-coated catheters.** The Journal of Vascular Access, 16 Suppl 9, S48-53. https://doi.org/https://dx.doi.org/10.5301/jva.5000376

Although tunneled hemodialysis catheter must be considered the last option for vascular access, it is necessary in some circumstances in the dialysis patient. Thrombosis and infections are the main causes of catheter-related comorbidity. Fibrin sheath, intimately related with the biofilm, is the precipitating factor of this environment, determining catheter patency and patient morbidity. Its association with bacterial overgrowth and thrombosis has led to the search of multiple preventive measures. Among them is the development of catheter coatings to prevent thrombosis and infections. There are two kinds of treatments to cover the catheter surface: antithrombotic and antimicrobial coatings. In nondialysis- related settings, mainly in intensive care units, both have been shown to be efficient in the prevention of catheter- related infection. This includes heparin, silver, chlorhexidine, rifampicine and minocycline. In hemodialysis population, however, few studies on surface-treated catheters have been made and they do not provide evidence that shows complication reduction. The higher effectiveness of coatings in nontunneled catheters may depend on the short average life of these devices. Hemodialysis catheters need to be used over long periods of time and require clinical trials to show effectiveness of coatings over long periods. This also means greater knowledge of biofilm etiopathogeny and fibrin sheath development.

26. Heidari Gorji, M. A., Rezaei, F., Jafari, H., & Yazdani Cherati, J. (2015). **Comparison of the effects of heparin and 0.9% sodium chloride solutions in maintenance of patency of central venous catheters.** Anesthesiology and Pain Medicine, 5(2), e22595. https://doi.org/https://dx.doi.org/10.5812/aapm.22595
BACKGROUND: Occlusion of central venous catheters is one of the limiting factors in using them. Heparinized saline solution is the standard solution used for keeping the catheters open., OBJECTIVES: This study aimed to determine the effect of heparin saline solution and normal saline in maintenance of patency of central venous catheters., PATIENTS AND METHODS: This double-blind study was performed on 84 patients of intensive care unit who had central venous catheters. The patients were randomly divided into two groups of heparin saline receivers and normal saline receivers. In the heparin group after each drug injection into the lumen, 3 mL of heparin saline solution was injected in the catheter as well. The other group only received 10 mL of normal saline instead. The catheters were examined for blood return and flushing every eight hours for 21 days. Data was analyzed using SPSS software version 20 and descriptive and analytic statistics were studied., RESULTS: There was no significant difference in the rate of flushing (P = 0.872) and possibility of taking blood samples from catheters (P = 0.745) in the two groups of heparin and normal saline receivers. Furthermore, using heparin had no effect on prolonging the survival of catheters., CONCLUSIONS: Considering possible side effects of heparin and the increase in treatment charges and the fact that using heparin did not have a significant effect on patency and survival of catheters in the studied patients, it is recommended to use normal saline solution to maintain the patency of central venous catheters.

27. R., B., A., S.-F., G., C., C., B., A., N., S., T., ... Quenot, J.-P. (2014). **Comparison of heparin to citrate as a catheter locking solution for non-tunneled central venous hemodialysis catheters in patients requiring renal replacement therapy for acute renal failure (VERROU-REA study): study protocol for a randomized controlled t**. Trials, 15(1), 449. https://doi.org/http://dx.doi.org/10.1186/1745-6215-15-449
BACKGROUND: The incidence of acute kidney injury (AKI) is estimated at 10 to 20% in patients admitted to intensive care units (ICU) and often requires renal replacement therapy (RRT). ICU mortality in AKI patients can exceed 50%. Venous catheters are the preferred vascular access method for AKI patients requiring RRT, but carry a risk of catheter thrombosis or infection. Catheter lock solutions are commonly used to prevent such complications. Heparin and citrate locks are both widely used for tunneled, long-term catheters, but few studies have compared citrate versus heparin for patients with short-term, non-tunneled catheters. We aim to compare citrate 4% catheter lock solution versus heparin in terms of event-free survival of the first non-tunneled hemodialysis catheter inserted in ICU patients with AKI requiring RRT. Secondary

objectives are the rate of fibrinolysis, incidence of catheter thrombosis and catheter-related infection per 1,000 catheter days, length of stay in ICU and in-hospital and 28-day mortality., METHODS/DESIGN: The VERROU-REA study is a randomized, prospective, multicenter, double-blind, parallel-group, controlled superiority study carried out in the medical, surgical and nephrological ICUs of two large university hospitals in eastern France. A catheter lock solution composed of trisodium citrate at 4% will be compared to unfractionated heparin at a concentration of 5,000 IU/mL. All consecutive adult patients with AKI requiring extracorporeal RRT, and in whom a first non-tunneled catheter is to be inserted by the jugular or femoral approach, will be eligible. Catheters inserted by the subclavian approach, patients with acute liver failure, thrombopenia or contraindication to systemic anticoagulation will be excluded. Patients will be followed up daily in accordance with standard practices for RRT until death or discharge., DISCUSSION: Data is scarce regarding the use of non-tunneled catheters in the ICU setting in patients with AKI. This study will provide an evidence base for recommendations regarding the use of anticoagulant catheter locks for the prevention of dysfunction in non- tunneled hemodialysis catheters in patients with AKI in critical or intensive care., TRIAL REGISTRATION: Registered with Clinicaltrials.gov (registration number: NCT01962116) on 27 August 2013.

28. Branas, P., Morales, E., Rios, F., Sanz, F., Gutierrez, E., Quintanilla, N., ... Chaves, F. (2014). **Usefulness of endoluminal catheter colonization surveillance cultures to reduce catheter-related bloodstream infections in hemodialysis.** American Journal of Infection Control, 42(11), 1182–1187. https://doi.org/https://dx.doi.org/10.1016/j.ajic.2014.07.022
BACKGROUND: To evaluate the use of surveillance cultures (SCs) to prevent catheter-related bloodstream infections (CRBSIs) in asymptomatic hemodialysis (HD) patients., METHODS: In 2011-2012, we conducted a prospective study of HD patients with tunneled cuffed central venous catheters (TCCs). Colonization of the catheter lumen was assessed every 15 days by inoculating ~5 mL endoluminal blood into aerobic culture bottles. Individual patients were triaged based on SC results: group 1 (negative); group 2 (coagulase-negative Staphylococcus [CoNS] with time-to-positivity (TTP) >14 hours); group 3 (CoNS with TTP <=14 hours); and group 4 (any microorganism other than CoNS and any TTP)., RESULTS: We studied 104 patients (129 TCCs). Median follow-up was 262.5 days (interquartile range [IR], 135.0- 365.0). A total of 1,734 SCs were collected (median, 18 per patient; IR, 10.0-24.0), of which 1,634 (94.2%) were negative (group 1) and 100 (5.8%) were positive (group 2: 79; group 3: 12, group 4: 9). In groups 2 and 3, 19 TCCs required antibiotic lock therapy (ALT). In group 4, all patients received intravenous therapy and ALT. Under this protocol, there were 0.27 episodes of CRBSI per 1,000 catheter days compared with 1.65 (P < .001) prior to its implementation., CONCLUSION: SCs based on easily accessible samples proved useful in triaging HD patients at a high risk of infection. Copyright © 2014 Association for Professionals in Infection Control and Epidemiology, Inc. Published by Elsevier Inc. All rights reserved.

29. B.P., M., & Jaksic T. (2012). **Pediatric Intestinal Failure and Vascular Access**. Surgical Clinics of North America, 92(3), 729–743.
https://doi.org/http://dx.doi.org/10.1016/j.suc.2012.03.012
Emerging developments in the care of intestinal failure (IF) patients have drastically improved their overall prognosis, with recently reported survival rates over 90%. IF patients remain an extremely complex population who benefit from specialized, multidisciplinary care. Advances in the provision of parenteral and enteral nutrition, progress in the management of IF-associated liver disease with parenteral fish oil and catheter-associated blood stream infection with ethanol lock therapy, and the availability of novel surgical interventions, such as the serial transverse enteroplasty procedure, have made this a dynamic health care field with the promise of ongoing improvements in outcomes for these patients. © 2012 Elsevier Inc..

30. M.E., S., D., P., C., S., S.T., M., Skrupky L P, Schallom, M. E., ... Skrupky, L. P. (2012). **Heparin or 0.9% sodium chloride to maintain central venous catheter patency: A randomized trial**. Critical Care Medicine, 40(6), 1820– 1826. https://doi.org/https://dx.doi.org/10.1097/CCM.0b013e31824e11b4
Objective: To compare heparin (3 mL, 10 units/mL) and 0.9% sodium chloride (NaCl, 10 mL) flush solutions with respect to central venous catheter lumen patency. Design(s): Single-center, randomized, open label trial. Setting(s): Medical intensive care unit and Surgical/Burn/Trauma intensive care unit at Barnes-Jewish Hospital, St. Louis, MO. Patient(s): Three hundred forty-one patients with multilumen central venous catheters. Patients with at least one lumen with a minimum of two flushes were included in the analysis. Intervention(s): Patients were randomly assigned within 12 hrs of central venous catheter insertion to receive either heparin or 0.9% sodium chloride flush. Measurements and Main Results: The primary outcome was lumen nonpatency. Secondary outcomes included the rates of loss of blood return, inability to infuse or flush through the lumen (flush failure), heparin-induced thrombocytopenia, and catheter-related blood stream infection. Assessment for patency was performed every 8 hrs in lumens without continuous infusions for the duration of catheter placement or discharge from intensive care unit. Three hundred twenty-six central venous catheters were studied yielding 709 lumens for analysis. The nonpatency rate was 3.8% in the heparin group (n = 314) and 6.3% in the 0.9% sodium chloride group (n = 395) (relative risk 1.66, 95% confidence interval 0.86-3.22, p = .136). The Kaplan-Meier analysis for time to first patency loss was not significantly different (log rank = 0.093) between groups. The rates of loss of blood return and flush failure were similar between the heparin and 0.9% sodium chloride groups. Pressure-injectable central venous catheters had significantly greater rates of nonpatency (10.6% vs. 4.3%, p = .001) and loss of blood return (37.0% vs. 18.8%, p <.001) compared to nonpressure-injectable catheters. The frequencies of heparin-induced thrombocytopenia and catheter-related blood stream infection were similar between groups. Conclusion(s): 0.9% sodium chloride and heparin flushing solutions have similar rates of lumen nonpatency. Given potential safety concerns with the use of heparin, 0.9% sodium chloride may be the preferred flushing solution for short-term use central venous catheter maintenance. © 2012 by the Society of Critical Care Medicine and Lippincott Williams & Wilkins.

31. N., M., A., L., J.-F., T., Souweine B, Mrozek, N., Lautrette, A., ... Souweine, B. (2012). **How to deal with dialysis catheters in the ICU setting.** Annals of Intensive Care, 2(1), 48. https://doi.org/https://dx.doi.org/10.1186/2110- 5820-2-48
Acute kidney insufficiency (AKI) occurs frequently in intensive care units (ICU). In the management of vascular access for renal replacement therapy (RRT), several factors need to be taken into consideration to achieve an optimal RRT dose and to limit complications. In the medium and long term, some individuals may become chronic dialysis patients and so preserving the vascular network is of major importance. Few studies have focused on the use of dialysis catheters (DC) in ICUs, and clinical practice is driven by the knowledge and management of long-term dialysis catheter in chronic dialysis patients and of central venous catheter in ICU patients. This review describes the appropriate use and management of DCs required to obtain an accurate RRT dose and to reduce mechanical and infectious complications in the ICU setting. To deliver the best RRT dose, the length and diameter of the catheter need to be sufficient. In patients on intermittent hemodialysis, the right internal jugular insertion is associated with a higher delivered dialysis dose if the prescribed extracorporeal blood flow is higher than 200 ml/min. To prevent DC colonization, the physician has to be vigilant for the jugular position when BMI < 24 and the femoral position when BMI > 28. Subclavian sites should be excluded. Ultrasound guidance should be used especially in jugular sites. Antibiotic-impregnated dialysis catheters and antibiotic locks are not recommended in routine practice. The efficacy of ethanol and citrate locks has yet to be demonstrated. Hygiene procedures must be respected during DC insertion and manipulation.

32. Sona, C., Prentice, D., Schallom, L., C., S., D., P., & Schallom L. (2012). **National survey of central venous catheter flushing in the intensive care unit**. Critical Care Nurse, 32(1), e12-9. https://doi.org/https://dx.doi.org/10.4037/ccn2012296
Evidence is needed on the best solution for flushing central venous catheters. To understand current flushing practices for short-term central venous catheters among critical care nurses before implementation of a randomized, controlled trial comparing physiological saline with heparin solution for flushing to maintain catheter patency. A 6-item survey including demographic data was mailed to 2000 practicing critical care nurses in the United States. An additional 316 surveys were completed at the annual conference of the American Association of Critical-Care Nurses. Most (71.5%) of the 632 respondents who completed the survey were staff nurses. Most respondents (64.6%; 95% CI, 60.86%-68.34%) reported using physiological saline exclusively to flush central venous catheters and maintain patency. For heparin- containing solutions, the concentration and volume used varied. The most commonly reported volumes for flushing were 10 mL for saline (63%; 95% CI, 59.18%-66.82%) and 3 mL for heparin (50.2%; 95% CI, 43.5%-56.9%). Flushing practices for central venous catheters vary widely. A randomized controlled trial is needed to determine the optimal flushing solution to maintain short-term patency.

33. L., H., J.-P., Q., A., N., S.D., B., P.-E., C., M., H., ... Freysz, M. (2012). **Sodium citrate versus saline catheter locks for non-tunneled hemodialysis central venous catheters in critically ill adults: A randomized controlled trial**. Intensive Care Medicine, 38(2), 279–285. https://doi.org/https://dx.doi.org/10.1007/s00134-011- 2422-y
PURPOSE: Sodium citrate has antibacterial and anticoagulant properties that are confined to the catheter when used as a catheter lock. Studies of its use as a catheter lock in chronic hemodialysis patients suggest it may be efficacious in preventing infection and thrombotic complications. We compared sodium citrate with saline catheter locks for non- tunneled hemodialysis central venous catheters in critically ill adult patients. Primary endpoint was catheter life span without complication., METHODS: This was a randomized, controlled, open-label trial involving intensive care patients with acute renal failure requiring hemodialysis. Events were defined as catheter-related bloodstream infection and catheter malfunction., RESULTS: Seventy-eight patients were included. Median catheter life span without complication was 6 days (saline group) versus 12 days (citrate group) [hazard ratio (HR) 2.12 (95% CI 1.32-3.4), p = 0.0019]. There was a significantly higher rate of catheter malfunction in the saline group compared with in the citrate group (127 catheter events/1,000 catheter-days, saline group vs. 26 events/1,000 catheter-days, citrate group, p < 0.00001). There was no significant difference in incidence of infections between groups. We observed a significantly longer time to occurrence of infection in the citrate group (20 days vs. 14 days, HR 2.8, 95% CI 1.04-7.6, p = 0.04). By multivariate analysis, age and citrate group were the only independent factors that influenced catheter life span., CONCLUSIONS: This study shows for the first time that citrate lock reduced catheter complications and increased catheter life span as compared to saline lock in critically ill adults requiring hemodialysis.

34. D.G., M., S.R., A., R.K., W., & Lavin P. (2011). **A novel antimicrobial and antithrombotic lock solution for hemodialysis catheters: A multi-center, controlled, randomized trial**. Critical Care Medicine, 39(4), 613–620. https://doi.org/http://dx.doi.org/10.1097/CCM.0b013e318206b5a2
Background and Purpose: Catheter-related bloodstream infection is the greatest threat to the safety of patients on hemodialysis. Catheter lock solutions containing heparin have been linked to an increased risk of hemorrhage and thrombocytopenia. Objective(s): To ascertain the safety and efficacy for prevention of catheter-related bloodstream infection and catheter loss from patency

failure of a novel catheter lock solution with antimicrobial and antithrombotic activity containing 0.24 M (7.0%) sodium citrate, 0.15% methylene blue, 0.15% methylparaben, and 0.015% propylparaben (C-MB-P), compared with heparin. Design(s): Multicenter, prospective, randomized, open-label trial with patients studied for up to 6 months. An independent clinical evaluation committee assessing trial outcomes was blinded to patients treatment assignments. Setting(s): Twenty-five outpatient hemodialysis units. Patient(s): Patients with end- stage renal disease receiving maintenance hemodialysis through a percutaneous cuffed and tunneled internal jugular hemodialysis catheters. Intervention(s): Participants catheters were locked between hemodialysis sessions with the C- MB-P lock solution or sterile saline containing 5000 units of unfractionated heparin (control). Measurements and Main Results: We recorded and evaluated catheter-related bloodstream infections, catheter loss attributable to luminal thrombosis, and adverse events. A total of 407 patients participated in the trial (49,565 catheter days), 201 in the C-MB- P group and 206 in the heparin group. Patients in the two lock solution groups were comparable for risk factors predisposing to catheter-related bloodstream infection. Catheters locked with C-MB-P were significantly less likely to cause catheter-related bloodstream infection (0.24 vs. 0.82 per 1000 catheter days; relative risk, 0.29; 95% confidence interval, 0.12-0.70; p = .005) and were less likely to be lost because of patency failure (0 vs. 4; log rank, p = .04). Conclusion(s): The novel C-MB-P lock solution is well tolerated, significantly reduces the risk of catheter-related bloodstream infection, and provides protection comparable to heparin against patency failure. Copyright © 2011 by the Society of Critical Care Medicine and Lippincott Williams & Wilkins.

35. Mathers D. (2011). **Evidence-based practice: Improving outcomes for patients with a central venous access device**. JAVA - Journal of the Association for Vascular Access, 16(2), 64–72. https://doi.org/http://dx.doi.org/10.2309/java.16-2-3
Purpose: To apply evidence-based practice when flushing central venous access devices (CVADs). Background/Introduction: There is a lack of standardized flushing protocols for CVADs among health care institutions. Identifying best practice and assuring skillful implementation of evidence-based practice protocols is essential to maintaining catheter patency. Review of Relevant Literature: Evidence supports the use of nonheparinized saline flush, positive-pressure valve caps, and proper flushing technique to maintain CVAD patency (Bowers, Speroni, Jones, & Atherton, 2008; Hadaway, 2006; & Jasinsky & Wurster, 2009). Reinforcement of proper flushing techniques has demonstrated improved patency rates of CVADs (Feehery, Allen, & Bey, 2003). Method(s): Corporate and individual in- services were conducted to improve nurses' skill and knowledge of evidence-based practice related to flushing CVADs. Outcomes were measured by comparing baseline data with data collected in the same manner post-education. Data was obtained by means of a questionnaire and direct observation of nurses' flushing technique. Outcome(s): The evidence- based practice project demonstrated a significant improvement (p <0.05) in both the nurses' knowledge and skill in flushing CVADs. Conclusion(s): Continuing education and reinforcement of proper flushing technique is an appropriate strategy to increase knowledge of and compliance with evidence-based practice protocols. Implications for practice: Providing continuing education and periodic reinforcement of nursing skills can lead to improved patient outcomes. These strategies, along with changing flushing protocols to non-heparinized saline, can also reduce health care costs. Future studies are needed to determine the appropriate frequency of in-service education.

36. P., C., Sherertz R J, Chittick, P., & Sherertz, R. J. (2010). **Recognition and prevention of nosocomial vascular device and related bloodstream infections in the intensive care unit.** Critical Care Medicine, 38(8 Suppl), S363- 72.
https://doi.org/https://dx.doi.org/10.1097/CCM.0b013e3181e6cdca
Central venous catheters have become a mainstay in the care of critically ill patients but, unfortunately, are associated with a significant risk of bloodstream infections. There are 80,000 catheter-related bloodstream infections that occur annually in the United States, with a high human and financial cost. This paper reviews the main tools for prevention and diagnosis of

central venous catheter-related bloodstream infections in the intensive care unit. We discuss specific aspects of prevention, including education, hand hygiene, sterile technique, skin cleansing, choice of catheter site, antimicrobial-impregnated catheters, catheter site dressings, antibiotic lock solutions, anticoagulation, catheter changes, and needleless connection devices. An analysis of studies evaluating the use of catheter "bundles" is also included. Diagnostic methods discussed include how to obtain blood cultures, when to culture catheter tips, how to interpret culture results, and the best methods for diagnosis.

37. Jonker, M. A., Osterby, K. R., Vermeulen, L. C., Kleppin, S. M., Kudsk, K. A., M.A., J., ... Kudsk K A. (2010). **Does low-dose heparin maintain central venous access device patency? A comparison of heparin versus saline during a period of heparin shortage**. Journal of Parenteral and Enteral Nutrition, 34(4), 444–449. https://doi.org/https://dx.doi.org/10.1177/0148607110362082
BACKGROUND: A common problem that complicates use of central venous access devices (CVADs) is occlusion by thrombosis. Alteplase, a recombinant tissue plasminogen activator, is used to restore line patency when thrombosis occurs. Heparin flush is commonly used to prevent this complication, but the effectiveness of this practice is unclear. A recent heparin shortage allowed examination of heparin effectiveness in reducing CVAD thrombosis., METHODS: A retrospective cohort study was performed by querying a pharmacy database for alteplase use for CVAD thrombosis in adult patients during periods when heparin flushes (10 units/mL) were used and when saline flushes were used instead because of a nationwide heparin shortage. The number of patients receiving alteplase, the number of doses administered, and the total amount of alteplase used were compared over 1-month intervals of heparin flush use and 1- month intervals of saline flush use. Patient days and critical care patient days were compared between these time intervals. Peripherally inserted central catheter (PICC) line placements and replacements between time periods of heparin and saline flush were also compared., RESULTS: Significant increases in the number of patients receiving alteplase (P = .04), the number of alteplase doses administered (P = .04), and total dose of alteplase used (P = .05) occurred during the heparin shortage. No significant differences in patient population were observed. The percentage of PICC line replacements also increased significantly (P < .05) when heparin was not available., CONCLUSIONS: Heparin flush (10 units/mL) decreases thrombotic occlusions of CVADs, resulting in decreased alteplase use and fewer PICC line replacements.

Literature Search Results

Date Completed: 2022-11-28 **Requestor:** Marlena Ornowska

Request: UPDATE: Efficacy of Various Locking Solutions in the ICU- Systematic Review project with Dr. Steve Reynolds

Research Question: What is the effectiveness of various central venous access device (CVAD) locking solutions in preventing complications modifiable by CVAD locking compared to standard of care saline locking fluids (as described by a statistically significant difference between groups)?

Population: Adult (>18 years old) Intensive Care Unit patients in North America and Europe. **Intervention/Exposure:** various CVAD locking solutions- antibiotic, heparin, ethanol, citrate, EDTA, taurolidine. **Comparisons:** Compared to standard of care- saline locking solutions.

Outcomes: Any of the following- CLABSI and/or CRBSI, bloodstream infection, bacterial biofilm formation, occlusion, catheter-related thrombosis (symptomatic and asymptomatic), alteplase use, local infection/phlebitis, premature catheter replacement, length of ICU stay, length of hospital stay, mortality.

Inclusion/Exclusion Criteria:

- - Papers meeting the review objectives described above
- - Years Considered: must be published within the last 2 years (2020-2022).
- - Language considered: English
- - Publication status: published in a peer-reviewed journal

Exclude: Case reports, posters, conference abstracts. **Completed By:** Sarah Gleeson Noyes

Search Strategy:

Ovid MEDLINE(R) and In-Process & Other Non-Indexed Citations 1946 to November 23, 2022

1. Central Venous Catheters/
2. Catheterization, Central Venous/
3. Catheter* adj2 central.ti,ab,kw.
4. Central venous access device*.ti,ab,kw.
5. OR/1-4
6. Lock*.ti,ab,kw.
7. Patency.ti,ab,kw.
8. OR/6-7
9. Exp Intensive Care Units/ 10. Exp Critical Care/
11. ICU.ti,ab,kw.
12. Intensive care.ti,ab,kw. 13. Critical care.ti,ab,kw.
14. OR/9-13
15. AND/5,8,14
16. Child*.ti,ab,kw.
17. Neonat*.ti,ab,kw.
18. OR/16-17
19. 15 NOT 18

[Limits: no letters, comments or case reports; 2020-present; English language]

Results from Medline only: 4

EMBASE 1996 to 2022 Week 46
1. Central Venous Catheter/
2. Central Venous Catheterization/
3. Catheter* adj2 central.ti,ab,kw.
4. Central venous access device*.ti,ab,kw.
5. OR/1-4
6. Lock*.ti,ab,kw.Patncy.ti,ab,kw.

7. OR/6-7
8. Exp Intensive Care Unit/
9. Intensive Care/
10. ICU.ti,ab,kw.
11. Intensive care.ti,ab,kw.
12. Critical care.ti,ab,kw.
13. OR/9-13
14. AND/5,8,14
15. Child*.ti,ab,kw.
16. Neonat*.ti,ab,kw.
17. OR/16-17
18. 15 NOT 18

[Limits: no letters or conference abstracts; 2020-present; English language]

Results from EMBASE only: 3

Total De-duplicated Results: 5

1. Lopez-Pena G., Anaya-Ayala J.E., Garcia-Alva R., Luna L., Lizola R., Cuen-Ojeda C., ... Hinojosa C.A. (2020). **Comparison of axillary-atrial and axillary-iliac arteriovenous grafts for hemodialysis access creation**. Journal of Vascular Access, 21(1), 55–59. https://doi.org/10.1177/1129729819851919
Objective: The aim of this study was to compare two complex vascular access techniques that utilize the axillary artery as inflow and accesses were created with early cannulation grafts: the axillary-atrial arteriovenous graft versus axillary-iliac arteriovenous graft. Method(s): This is a retrospective study of end-stage renal disease patients with occluded intrathoracic central veins that underwent complex hemodialysis access creation in our institution after failed endovascular recanalization attempts. Patients' demographics, comorbidities, number and types of previous accesses, intraoperative variables, and clinical outcomes were collected and compared. Result(s): Four patients underwent axillary-atrial arteriovenous graft creation with FlixeneTM (AtriumTM, Hudson, NH, USA) grafts, through a midline sternotomy to expose the right atrium; all were successfully implanted and used for hemodialysis within the first 72 h; one patient developed a pseudoaneurysm in the mid- graft portion, requiring surgical repair, and it is currently functional. Eight axillary-iliac arteriovenous grafts were created; all grafts were patent and were utilized within 96 h after placement. At 6 months of follow-up period, five (62 %) of our patients underwent graft thrombectomy, one (12 %) balloon angioplasty at the vein anastomosis secondary to stenosis, and two (25 %) grafts were removed due to infectious complications. Axillary-atrial arteriovenous graft and axillary-iliac arteriovenous graft primary patency rates at 6 months were 75% and 48%, respectively; 6-month secondary patency of the axillary-atrial arteriovenous graft compares favorably against that of axillary-iliac arteriovenous graft (100% vs 75%, respectively). Conclusion(s): Despite the invasiveness, direct atrial outflow procedures remain a valid alternative in carefully selected patients with adequate cardiopulmonary reserve.Copyright © The Author(s) 2019.

2. Zamir, N., Pook, M., McDonald, E., & Fox-Robichaud, A. E. (2020). **Chlorhexidine locking device for central line infection prevention in ICU patients: Protocol for an open-label pilot and feasibility randomized controlled trial.** Pilot and Feasibility Studies, 6(101676536), 26.
https://doi.org/10.1186/s40814-020-0564-9
BACKGROUND: Critically ill patients in the intensive care unit (ICU) are at risk for central line-associated bloodstream infection (CLABSI) with an incidence up to 6.9 per 1000 catheter days. CLABSI has a significant attributable mortality and increases in- hospital length of stay, readmissions, and costs. Chlorhexidine gluconate (CHG), a broad-spectrum biocide, has been shown to effectively reduce infections including CLABSI; however, few trials have utilized CHG for prevention of central line infections. Our preclinical work has demonstrated a device that diffuses CHG into the intravenous lock solution of central venous catheters and decreases bacterial growth on the catheter lumen. We designed a clinical trial to test the feasibility of using a CHG device in an ICU patient population., METHODS: The proposed pilot trial will be a single centre, open-label, two-arm, parallel group feasibility randomized controlled trial (RCT). Participants will have a central line in situ and will be enrolled within 72 h of admittance to 3 ICUs at a single academic hospital. Exclusion criteria will include suspected infection, chronic indwelling catheters, and CHG allergy. Informed consent will be obtained from eligible participants or their substitute decision maker prior to randomization. Participants will be randomized to receive either usual care or the CHG locking device. Blood cultures will be drawn from all participants every 48 h. The primary objective of this study will be to determine the feasibility of using this protocol to conduct a larger trial. Feasibility will be assessed through the following outcomes: (1) consent rate, (2) recruitment rate, (3) protocol adherence, and (4) comfort level with the device. The secondary objective of this study will be to establish the preliminary efficacy of the device., DISCUSSION: This study will be the first human RCT to investigate a CHG locking device for the prevention of central line infections. Findings from this trial will inform the feasibility of conducting a large RCT and provide preliminary data on the efficacy of a CHG locking device., TRIAL REGISTRATION: ClinicalTrials.gov, NCT03309137, registered on October 13, 2017. Copyright © The Author(s). 2020.

3. Yu, H., Xin, Q., Wang, X., Jia, L., Wang, J., Meng, X., ... Jiang, A. (2020). **Effects of different catheter replacement methods on catheter service time and complications in hemodialysis patients: A cohort study.** The Journal of Vascular Access, 21(4), 497–503.
https://doi.org/10.1177/1129729819891336
INTRODUCTION: Central venous catheter insertion for long-term vascular access is not recommended in clinical practice. However, since arteriovenous fistula creation is difficult to perform in some patients, central venous catheter insertion for long-term vascular access is performed. This study aimed to assess the complications and service time of central venous catheters replaced using different methods and to determine the influencing factors of service time., METHODS: Study design: A retrospective observational cohort study. Setting and participants: Patients who underwent tunneled dialysis catheter malfunction (2009-2019) and had to undergo another dialysis catheter insertion were enrolled. Exposures: Ectopic replacement and in situ replacement. Outcomes: Factors such as age, sex, primary patency rate, secondary patency rate, early complications, and late complications were considered. Analytical approach: This study used a Cox proportional hazards regression model., RESULTS: The first and the newly replaced catheter service time were 37.779 +/- 24.563 months and 32.468 +/- 26.638 (25) months in the ectopic group and 37.075 +/- 20.550 months and 26.349 +/- 22.672 months in the in situ group, respectively. In the early service time, the newly replaced catheter resulted in significant bleeding from the tunnel. The first catheter had the least complications, most adequate blood flow, and longest service time. Ectopic catheter replacement and the tip shape of the catheter were the independent factors for catheter service time. Catheter service time increased with age., CONCLUSION: Ectopic catheter replacement can improve the primary patency rate and auxiliary primary patency rate of catheters. Ectopic catheter replacement may require sufficient surgical skills with digital subtraction angiography, resulting in a better prognosis.

4. Javeri, Y., Jagathkar, G., Dixit, S., Chaudhary, D., Zirpe, K. G., Mehta, Y., ... Jain, R. (2020). **Indian Society of Critical Care Medicine Position Statement for Central Venous Catheterization and**

Management 2020. Indian Journal of Critical Care Medicine : Peer-Reviewed, Official Publication of Indian Society of Critical Care Medicine, 24(Suppl 1), S6–S30. https://doi.org/10.5005/jp-journals-10071-G23183

BACKGROUND AND PURPOSE: Short-term central venous catheterization (CVC) is one of the commonly used invasive interventions in ICU and other patient-care areas. Practice and management of CVC is not standardized, varies widely, and need appropriate guidance. Purpose of this document is to provide a comprehensive, evidence-based and up-to-date, one document source for practice and management of central venous catheterization. These recommendations are intended to be used by critical care physicians and allied professionals involved in care of patients with central venous lines., METHODS: This position statement for central venous catheterization is framed by expert committee members under the aegis of Indian Society of Critical Care Medicine (ISCCM). Experts group exchanged and reviewed the relevant literature. During the final meeting of the experts held at the ISCCM Head Office, a consensus on all the topics was made and the recommendations for final document draft were prepared. The final document was reviewed and accepted by all expert committee members and after a process of peer-review this document is finally accepted as an official ISCCM position paper.Modified grade system was utilized to classify the quality of evidence and the strength of recommendations. The draft document thus formulated was reviewed by all committee members; further comments and suggestions were incorporated after discussion, and a final document was prepared., RESULTS: This document makes recommendations about various aspects of resource preparation, infection control, prevention of mechanical complication and surveillance related to short-term central venous catheterization. This document also provides four appendices for ready reference and use at institutional level., CONCLUSION: In this document, committee is able to make 54 different recommendations for various aspects of care, out of which 40 are strong and 14 weak recommendations. Among all of them, 42 recommendations are backed by any level of evidence, however due to paucity of data on 12 clinical questions, a consensus was reached by working committee and practice recommendations given on these topics are based on vast clinical experience of the members of this committee, which makes a useful practice point. Committee recognizes the fact that in event of new emerging evidences this document will require update, and that shall be provided in due time., ABBREVIATIONS LIST: ABHR: Alcohol-based hand rub; AICD: Automated implantable cardioverter defibrillator; BSI: Blood stream infection; C/SS: CHG/silver sulfadiazine; Cath Lab: Catheterization laboratory (Cardiac Cath Lab); CDC: Centers for Disease Control and Prevention; CFU: Colony forming unit; CHG: Chlorhexidine gluconate; CL: Central line; COMBUX: Comparison of Bedside Ultrasound with Chest X-ray (COMBUX study); CQI: Continuous quality improvement; CRBSI: Catheter-related blood stream infection; CUS: Chest ultrasonography; CVC: Central Venous Catheter; CXR: Chest X-ray; DTTP: Differential time to positivity; DVT: Deep venous thrombosis; ECG: Electrocardiography; ELVIS: Ethanol lock and risk of hemodialysis catheter infection in critically ill patients; ER: Emergency room; FDA: Food and Drug Administration; FV: Femoral vein; GWE: Guidewire exchange; HD catheter: Hemodialysis catheter; HTS: Hypertonic saline; ICP: Intracranial pressure; ICU: Intensive Care Unit; IDSA: Infectious Disease Society of America; IJV: Internal jugular vein; IPC: Indian penal code; IRR: Incidence rate ratio; ISCCM: Indian Society of Critical Care Medicine; IV: Intravenous;

LCBI: Laboratory confirmed blood stream infection; M/R: Minocycline/rifampicin; MBI-LCBI: Mucosal barrier injury laboratory- confirmed bloodstream infection; MRSA: Methicillin-resistant Staphylococcus aureus; NHS: National Health Service (UK); NHSN: National Healthcare Safety Network (USA); OT: Operation Theater; PICC: Peripherally-inserted central catheter; PIV: Peripheral intravenous line; PL: Peripheral line; PVI: Povidone-iodine; RA: Right atrium; RCT: Randomized controlled trial; RR: Relative risk; SCV/SV: Subclavian vein; ScVO2: Central venous oxygen saturation; Sn: Sensitivity; SOP: Standard operating procedure; SVC: Superior vena cava; TEE: Transesophageal echocardiography; UPP: Useful Practice Points; USG: Ultrasonography; WHO: World Health Organization., HOW TO CITE THIS ARTICLE: Javeri Y, Jagathkar G, Dixit S, Chaudhary D, Zirpe KG, Mehta Y, et al. Indian Society of Critical Care Medicine Position Statement for Central Venous Catheterization and Management 2020. Indian J Crit Care Med 2020;24(Suppl 1):S6-S30. Copyright © 2020; Jaypee Brothers Medical Publishers (P) Ltd.

5. Bovet, J., Soudry-Faure, A., Merdji, H., Ksiazek, E., Quenot, J.-P., Meziani, F., ... Helms, J. (2020). **Evaluation of anti-Xa activity after injection of a heparin lock for dialysis catheters in intensive care: A prospective observational study.** Thrombosis Research, 188(vrn, 0326377), 82–84. https://doi.org/10.1016/j.thromres.2020.02.006
