# Supplementary material for: Locking solutions for prevention of central venous access device complications in the adult critical care population: A systematic review
Source: PLoS One. 2023 Oct 12;18(10):e0289938. doi: 10.1371/journal.pone.0289938 (PMC10569507; doi:10.1371/journal.pone.0289938)
Supplement: S2 File — (PDF) [file pone.0289938.s004.pdf]

Appendix II: Quality Appraisal Justifications for studies included in the systematic review

Schallom et al., 2012

Sources Reviewed:
In order to complete this quality appraisal, we reviewed the original study article and connected with study authors regarding: clarification of study procedures, additional details of the CRBSI outcome data collection and analysis. Study authors replied with all information necessary.

Domain:	Judgement:	Justification:
**Domain 1:** Risk of Bias Arising from Randomization Process	Low	Quote: “Randomization was performed according to the Consolidated Standards for the Reporting of Trials (CONSORT) guidelines. A random allocation sequence was created using a computerized random number generator in Microsoft Excel. Simple randomization was done such that consecutively numbered cards with the flush assignment according to the randomization schedule written on the back of each card. The allocation sequence was concealed until the card was retrieved upon obtaining patient consent.”<br><br>Results of all statistical tests show insignificant p-values and suggest no differences in baseline characteristics between intervention groups (as seen in Schallom et al. Table 2).
**Domain 2:** Risk of bias due to deviations from the intended interventions (effect of assignment to intervention)	Low	Quote: “The assigned flush solution was entered into the electronic medication administration record and a sign with flush assignment was posted at the bedside.”<br><br>Schallom et al., Figure 1 shows no evidence of deviations from the intended intervention that could have been caused by the open-label design. The majority of patients who were randomized received their intended treatment (Saline group: allocated n=166, received saline n= 165. Heparin group: allocated n=172, received heparin n= 167). All outcome analyses were performed according to the intention-to-treat principle.<br><br>The study discussion states that 4.5% of flushes were undocumented. Authors showed the quantity of undocumented flushes was not different between groups (p=0.213).
**Domain 3:** Risk of bias due to missing outcome data	Low	Quote: “There was no significant difference in missed [patency] assessments between flushing groups (p=0.213).”<br><br>Additionally, Schallom et al. Figure 1 indicates data for nearly all (>95%) randomized participants was included in the final analysis, for both CRBSI and occlusion outcomes.
Outcome 1: CRBSI<br>**Domain 4:** Risk of bias in measurement of the outcome	Low	From e-mail correspondence with study authors we learned that independent Infection Prevention Specialists ascertained any occurrences of CRBSI that occurred during the study. The National Healthcare Safety Network definition of CRBSI was used. Authors of the study state it is possible, but highly unlikely that the external specialists might have reviewed flush solution that the patient had received, thereby leading to potential unblinding. Despite the possibility of unblinded CRBSI ascertainment, we feel the CRBSI

			definition used in the study was objective enough to warrant little chance of potential unblinding influencing outcome adjudication.
	**Domain 5:** Risk of bias in selection of the reported result	Low	All statistical testing mentioned at the start of the paper was presented in the results section.
	**Overall Risk of Bias: Low**		
Outcome 2: Occlusion	**Domain 4:** Risk of bias in measurement of the outcome	Low	Quote: "[lumen nonpatency was] defined as inability to both with-draw blood and flush through a lumen. Arrival at this end point occurred only after completion of the following interventions with the patency assessment: 1) if the lumen could not be flushed, the patient was repositioned and flush reattempted; 2) if still unable to flush, the needleless access device was changed and flush was reattempted. If the nurse could neither obtain blood return nor flush the lumen after these maneuvers, the lumen met criteria for nonpatency."<br><br>Quote: "Daily at 8:00 AM, one of the nurse investigators conducted a patency assessment for all lumens without a continuous or pressurized infusion. Daily at 4:00 PM and 12:00 AM, the patient's bedside nurse performed the patency assessments and documented results on a data collection sheet located at the patient's bedside. Therefore, three patency assessments per lumen assessment day were possible. These assessments were performed daily until the CVC was removed or until 1 day after the patient was transferred out of the ICU, whichever came first."<br><br>We deem outcome definition as appropriate; method of outcome measurement did not differ between groups. Although outcome assessors were aware of the treatment allocation, method of measured is deemed objective and systematic enough for little risk of this biasing the result of nonpatency assessment.
	**Domain 5:** Risk of bias in selection of the reported result	Low	All statistical testing mentioned at the start of the paper was presented in the results section.
	**Overall Risk of Bias: Low**		

Hermite et al., 2012

Sources Reviewed:
In order to complete this quality appraisal, authors reviewed the original study article. The author was contacted in an attempt to gain additional information, however was unable to be reached despite multiple contact attempts.

Domain:	Judgement:	Justification:
**Domain 1:** Risk of Bias Arising from Randomization Process	Low	Quote: "All patients included in the study were randomized between the control group (saline group) and the experimental group (citrate group) (treatment allocation given in numbered sealed envelopes)."<br><br>Hermite et al. Table 1 shows no significant differences in baseline characteristics between groups.
**Domain 2:** Risk of bias due to deviations from the intended interventions (effect of assignment to intervention)	Low	Quote: "This was a prospective, randomized, open-label trial con- ducted from May 2009 to August 2010 in one medical and one surgical ICU […]"<br><br>Quote: "The study procedures were performed by research nurses, who were aware of the treatment allocation, but were not involved in routine care of the patient."<br><br>Quote: "As far as possible, the nurses and physicians in the unit in charge of the routine care were blinded to each patient's treatment."<br><br>Quote: "All analyses were performed by intention-to-treat."<br><br>Although information about initial patient allocation and receipt of intervention is missing from the paper, we adjudicate this paper to have a low risk of bias due to deviations from the intended interventions due to locking being performed by an external research nurse. As such, there is little evidence bias could have arisen based on the trial context.
**Domain 3:** Risk of bias due to missing outcome data	High	The paper states that 78 patients were included in the study, however does not give any information about the original number randomized and if any were lost to follow-up or otherwise censored for any reason as the study progressed. There was no information about the handling of missing data, and no information about sensitivity analysis or other analysis methods to account for missing data. Further, there is no information about differences between intervention groups in the proportions of missing data, and no information about reasons for missing data between groups. There was no information about the analysis accounting for any patient characteristics that are likely to explain missing data.
Outcome 1: CRBSI<br>**Domain 4:** Risk of bias in measurement of the outcome	Low	Quote: "Catheter-related bloodstream infection (CRBSI) was defined as fever (temperature[38°C) with concordant positive blood cultures drawn from the catheter and a peripheral vein or a peripheral blood culture and a concordant exit-site culture, in the absence of any other clinically identifiable source of bloodstream infection other than the catheter."<br><br>Quote: "Study patients were evaluated for fever and other signs suggestive of infection every 3h by the nurses on duty, according to standard practice in our unit. Blood cultures, exit-site cultures if there was purulence at the exit site, and other studies, as indicated, were obtained whenever study patients had fever or chills, unexplained hypotension, or

			other clinical signs or symptoms suggestive of systemic infection, as previously described.”<br><br>Quote: “Outcomes were evaluated locally and adjudicated by an independent clinical event committee composed of a nephrologist and an infectious disease specialist who were not involved in the trial and were blinded to patients’ treatment group assignments.”<br><br>There is no evidence to suggest the method of measurement or ascertainment of outcome differed between groups, nor that outcome assessors were aware of intervention received.
	**Domain 5:** Risk of bias in selection of the reported result	Low	All statistical testing that was introduced in the paper methodology was performed and reported on in the results section
	**Overall Risk of Bias: High**		
Outcome 2: Occlusion	**Domain 4:** Risk of bias in measurement of the outcome	High	Quote: “Catheter malfunction was thus defined as a reduction of 20% or more in blood flow through the catheter, despite attempts to restore patency.”<br><br>The method of measuring the outcome is appropriate.<br><br>Patient catheters were assessed for non-patency as nurses accessed the catheter for standard patient care. Authors state that once catheters were locked with citrate, all catheter manipulations were forbidden until the next hemodialysis session. This was not stated to be the case for catheters locked with saline. As such, it is possible that catheters were manipulated more frequently in the saline group, resulting in a more frequent monitoring of occlusion that may have led to overestimation of this outcome and a possible explanation of the significant result.<br><br>Data was not collected regarding the amount of times each CVAD might have been accessed in each group.
	**Domain 5:** Risk of bias in selection of the reported result	Low	All statistical testing that was introduced in the paper methodology was performed and reported on in the results section
	**Overall Risk of Bias: High**		

Souweine et al., 2015

Sources Reviewed:
Review authors reviewed the original study article and connected with study authors regarding clarification of study procedures as well as more details of the CRBSI and catheter dysfunction outcome data collection and analysis. Study authors replied with all information necessary.

Domain:		Judgement:	Support from text of the paper:
**Domain 1:** Risk of Bias Arising from Randomization Process		Low	Quote: “Randomization was done using a web-based random-number generator producing permuted blocks, with stratification on ICU.”<br><br>Quote: “The ethanol and 0.9% saline locks were identical in appearance. The solutions were colorless and provided in vials of identical shape and appearance…Concealment of allocation and provision of blinding was guaranteed by the French General Agency for Medicines and Health Products, which delivered patient-labeled vials containing either ethanol or 0.9% saline.”<br><br>Souweine et al. Table 1 shows no significant differences in baseline characteristics between groups.
**Domain 2:** Risk of bias due to deviations from the intended interventions (effect of assignment to intervention)		Low	Quote: “We conducted a multicenter, randomized, double-blind, placebo-controlled, parallel- group study in France.”<br><br>Quote: “An audit to look for evidence of unblinding showed that none of the healthcare workers was able to unmask the study locks. In addition to the patients, physicians, and nurses, the outcome assessors and data analysts were masked to the treatment group until validation of the final results.”<br><br>The primary analysis was performed via intention-to-treat, and results of both intention-to-treat and per protocol conditions were presented.<br><br>There is no evidence to suggest risk of bias due to deviations from the intended intervention.
**Domain 3:** Risk of bias due to missing outcome data		Low	Souweine et al. Figure 1 accounts for each patient as they pass through randomization, intention-to-treat, and per-protocol stages of the study and analysis. In each group, intention-to-treat data is available for >95% of patients randomized, and per-protocol data is available for 95% of patients randomized. This the same between groups.
Outcome 1: CRBSI	**Domain 4:** Risk of bias in measurement of the outcome	Low	Quote: “CRBSI was the presence of all three of the following criteria: one or more positive peripheral blood cultures sampled immediately before or within 48 hours after catheter removal, quantitative catheter-tip culture positive for the same microorganisms or blood-culture differential time-to-positivity of 2 hours or more, and no other site of infection explaining the positive blood cultures.”<br><br>Authors note that blinding was maintained until after full validation of data analysis was complete.<br><br>Outcome definition used was appropriate. There is no evidence to suggest the method of measurement or ascertainment of outcome differed between groups, nor that outcome assessors were aware of intervention received.

	**Domain 5:** Risk of bias in selection of the reported result	Low	All statistical testing that was introduced in the paper methodology was performed and reported on in the results section.
	**Overall Risk of Bias: Low**		
Outcome 2: Occlusion	**Domain 4:** Risk of bias in measurement of the outcome	Low	Quote: "Severe mechanical complications consisted of catheter obstruction or dysfunction that persisted despite attempts to restore patency and required catheter removal, and of catheter damage, such as split or lumen rupture."<br><br>After connecting with the corresponding author, we learned catheter dysfunction was assessed by nurses (sometimes by physicians) systematically at the start and end of dialysis sessions and when there were problems with the catheter flow and pressure. The nurses/physicians were blinded to the randomization status of the patient.<br><br>Authors note that blinding was maintained until after full validation of data analysis was complete.<br><br>Outcome definition used was appropriate. There is no evidence to suggest the method of measurement or ascertainment of outcome differed between groups, nor that outcome assessors were aware of intervention received.
	**Domain 5:** Risk of bias in selection of the reported result	Low	All statistical testing that was introduced in the paper methodology was performed and reported on in the results section
	**Overall Risk of Bias: Low**		
Outcome 3: Colonization	**Domain 4:** Risk of bias in measurement of the outcome	Low	Quote: "Catheter colonization was a positive quantitative catheter-tip culture (cutoffs, 1,000 CFU/ml with vortexing and 100 CFU/ml with sonication. When the catheter was not removed, it was considered colonized when a blood culture from the catheter hub was positive."<br><br>Authors note that blinding was maintained until after full validation of data analysis was complete.<br><br>Outcome definition used was appropriate. There is no evidence to suggest the method of measurement or ascertainment of outcome differed between groups, nor that outcome assessors were aware of intervention received.
	**Domain 5:** Risk of bias in selection of the reported result	Low	All statistical testing that was introduced in the paper methodology was performed and reported on in the results section
	**Overall Risk of Bias: Low**		

Quenot et al., 2019

Sources Reviewed:
In order to complete this quality appraisal, we reviewed the original study article, supplementary material, previously published protocol, and connected with study authors regarding clarification of handling of missing data. Study authors replied with all information necessary.

<table>
<tr><th colspan="2">Domain:</th><th>Judgement:</th><th>Support from text of the paper:</th></tr>
<tr><td colspan="2">Domain 1: Risk of Bias Arising from Randomization Process</td><td>Low</td><td>Quote: “Randomization was performed after verification of the inclusion and exclusion criteria via an online request using Tenalea software”

Quote: “The Inserm CIC- 1432 Clinical Epidemiology Unit (Dijon, France) managed the [randomization and allocation] data.”

Quote: “There was no significant difference in baseline characteristics between groups.”

Based on information presented in the paper, there is little evidence there any bias was introduced arising from the randomization process.</td></tr>
<tr><td colspan="2">Domain 2: Risk of bias due to deviations from the intended interventions (effect of assignment to intervention)</td><td>Low</td><td>Quote: “Since the study is a double-blind design, the investigators are unaware of the type of lock solution being instilled in the catheter for each patient.”

As stated in the paper, investigators and participants were unaware of the assigned intervention during the trial. The primary analysis was also based on the intention-to-treat principle.</td></tr>
<tr><td colspan="2">Domain 3: Risk of bias due to missing outcome data</td><td>Low</td><td>Quenot et al. Figure 1 accounts for each patient randomized as they complete the study. The intention-to-treat analysis includes 99% of patients originally randomized, and the per-protocol analysis includes 94%. The supplemental material also gives adequate and complete information about reasons patients were excluded from the study. As such there is low risk of bias introduced due to missing outcome data.</td></tr>
<tr><td>Outcome 1: CRBSI</td><td>Domain 4: Risk of bias in measurement of the outcome</td><td>Low</td><td>Quote: “Catheter-related blood stream infection, defined as the presence of fever (temperature > 38°C) and a positive blood culture taken from the dialysis catheter and a peripheral line in the absence of any other source of infection, with a central-to-peripheral quantitative blood culture ratio of over five, or a differential period of central line culture versus peripheral blood culture positivity for more than two hours, with a central hemoculture showing a positive result more quickly than a peripheral culture.”

Quote: “A clinical event committee comprising two physicians […] blinded to the treatment allocation will independently analyze data and adjudicate as to the presence or not of catheter infection.”

There is no evidence to suggest this outcome was measured inappropriately or differently between groups. As such, it is rated as having a low risk of bias.</td></tr>
</table>

	**Domain 5:** Risk of bias in selection of the reported result	Low	All of the results described in the methods section as well as the previously-published protocol are presented in the results section of the paper.
	**Overall Risk of Bias: Low**		
Outcome 2: Occlusion	**Domain 4:** Risk of bias in measurement of the outcome	Low	Quote: "Catheter dysfunction is defined as the inability to achieve and maintain a blood flow of more than 200 mL/min despite changing the patient's position, inverting the lines and flushing with saline solution. The occurrence of any one or more of these events (such as a change of position, inversion of a line or flush of saline solution) will be considered as catheter dysfunction." From Quenot et al. Table 3, we see this definition also includes withdraw of the CVAD. Review authors deem this definition as appropriate.<br><br>There is no evidence to suggest the outcome was measured differently between groups. As this was a double-blinded study, outcome assessors were unaware of the randomization status of the patient. Risk of bias in measurement of the outcome is determined to be low.
	**Domain 5:** Risk of bias in selection of the reported result	Low	All of the results described in the methods section as well as the previously-published protocol are presented in the results section of the paper.
	**Overall Risk of Bias: Low**		
Outcome 3: Colonization	**Domain 4:** Risk of bias in measurement of the outcome	Low	Quote: "**General** catheter-related infection, defined as a positive catheter-tip culture of 10^3 CFU/ml or more, with the disappearance of general signs of infection within 48 hours after catheter withdrawal." and "**Local** catheter-related infection, defined as a positive catheter-tip culture of 103 CFU/ml or more, with the presence of pus or oozing at the catheter insertion site, or tunnelitis."<br><br>Quote: "A clinical event committee comprising two physicians […] blinded to the treatment allocation will independently analyze data and adjudicate as to the presence or not of catheter infection."<br><br>There is no evidence to suggest this outcome was measured inappropriately or differently between groups. As such, it is rated as having a low risk of bias.
	**Domain 5:** Risk of bias in selection of the reported result	Low	All of the results described in the methods section as well as the previously-published protocol are presented in the results section of the paper.
	**Overall Risk of Bias: Low**		

Perez-Granda et al., 2014

Sources Reviewed:
In order to complete this quality appraisal, we reviewed the original study article, clinical trial registration, and connected with study authors regarding more details of allocation concealment, any deviations from the intervention, the 'catheter dysfunction requiring removal' outcome data collection, and analysis. We received no reply from corresponding authors despite a follow-up attempt.

Domain:		Judgement:	Support from text of the paper:
**Domain 1:** Risk of Bias Arising from Randomization Process		Some concerns	Quote: "Patients who gave their informed consent were randomly assigned into two groups: Ethanol group (E-lock) and conventional catheter-care (CCC)."<br><br>Information was presented in the paper regarding randomization used, however there was no mention of allocation concealment. Perez-Granda et al. Table 1 suggests no significant differences in baseline characteristics between groups. As such, review authors rate this study as having some concerns of risk of bias arising from the randomization process.
**Domain 2:** Risk of bias due to deviations from the intended interventions (effect of assignment to intervention)		Low	Quote: "This is an academic, prospective, randomized, non-blinded and controlled clinical trial […]."<br><br>Carers, people delivering the intervention, and participants of the study were aware of their group assignment.<br><br>Quote: "Only 2 patients failed to receive at least 1 dose of ethanol lock."<br><br>Although the reasons for deviation are not listed, due to the small number of deviations from the intended intervention, it is unlikely that this could have occurred due to the trial context or that is results in significant bias to the results. Results were analyzed according to an intention-to-treat analysis.
**Domain 3:** Risk of bias due to missing outcome data		Low	Quote: "The remaining 200 [randomized] patients constitute the Intention to Treat Population (ITT) and 198 the Per-Protocol Population (PP)."<br><br>Data from all 200 patients making up the intention to treat analysis is presented in Perez-Granda et al. Tables 2-5. There is low risk of bias resulting from missing outcome data.
Outcome 1: CRBSI	**Domain 4:** Risk of bias in measurement of the outcome	Some concerns	Quote: "The definitions of CRBSI are those detailed in the recent Clinical Practice Guidelines for the Diagnosis and Management of Intravascular Catheter-Related Infection. For the purpose of this study we only accepted microbiologically proven CRBSI considered when the same microorganism was recovered from blood and a catheter tip within less than 8 days."<br><br>This outcome definition is unclear as the reference document study authors refer to does not provide a clear definition of CRBSI. It is also unclear why the 8-day window was chosen.

			Quote: "For the purpose of this study, all removed catheters were sent for culture to the Microbiology Department (314 of 323)." Quote: "Surveillance cultures of the peri-catheter skin and hubs were systematically done until catheter removal." Data is presented for all patients included in the intention-to-treat group.<br><br>No information is given regarding blinding of outcome assessors. However, there is little to suggest that awareness of the treatment condition could have influenced assessment of the outcome. As such, this paper is rated as having some concerns of risk of bias in determination of the CRBSI outcome.
	**Domain 5:** Risk of bias in selection of the reported result	Low	The results of all pre-specified statistical tests are presented in the paper.
	**Overall Risk of Bias: Some concerns of risk of bias**		
Outcome 2: Occlusion	**Domain 4:** Risk of bias in measurement of the outcome	Low	Quote: "Obstruction of catheters that required catheter withdrawal occurred in 5 patients in the ethanol group and in 4 patients in the control group (p=0.87)"<br><br>Outcome definition of catheter obstruction was deemed to be appropriate. It is unclear whether any attempts to restore CVAD function (example: repositioning) were made prior to withdraw of the CVAD. There is no evidence to suggest this differed between groups. Although outcome assessors were likely aware of the randomization status of the patient, review authors feel the definition used is objective enough to not permit awareness of the treatment group to influence outcome assessment.
	**Domain 5:** Risk of bias in selection of the reported result	Low	The results of all pre-specified statistical tests are presented in the paper.
	**Overall Risk of Bias: Some concerns of risk of bias**		
Outcome 3: Colonization	**Domain 4:** Risk of bias in measurement of the outcome	Low	Quote: "Catheter tip colonization […] are defined as the presence of > or equal to15 colony forming units in the semiquantitative culture according to the roll plate technique recommendation"<br><br>Quote: "For the purpose of this study, all removed catheters were sent for culture to the Microbiology Department (314 of 323)." Quote: "Surveillance cultures of the peri-catheter skin and hubs were systematically done until catheter removal." Data is presented for all patients included in the intention-to-treat group.<br><br>Outcome definitions and methods of measurement are appropriate. No information is given regarding blinding of outcome assessors. However, there is little to suggest that awareness of the treatment condition could have influenced assessment of the outcome, or that assessment differed between treatment groups.
	**Domain 5:** Risk of bias in selection of the reported result	Low	The results of all pre-specified statistical tests are presented in the paper.
	**Overall Risk of Bias: Some concerns of risk of bias**		

Pook et al., 2022

Sources Reviewed:
In order to complete this quality appraisal, we reviewed the original study article, and previously published protocol.

<table>
<tr><th colspan="2">Domain:</th><th>Judgement:</th><th>Support from text of the paper:</th></tr>
<tr><td colspan="2">Domain 1: Risk of Bias Arising from Randomization Process</td><td>Low</td><td>Quote: “Randomization was conducted in blocks of two, four, six, and eight in a fixed 1:1 ratio using the statistical computer software, R v3.4.2. A third-party member used the list generated to prepare 100 opaque security envelopes containing a note that indicated either CHG or Standard Care. Upon obtaining informed consent, the research coordinator or assistant opened the security envelope that corresponded with the participant’s study ID to determine allocation.”

Allocation was concealed and appropriate randomization procedures were in place. Pook et al. Table 1 shows no statistically significant baseline differences between groups.</td></tr>
<tr><td colspan="2">Domain 2: Risk of bias due to deviations from the intended interventions (effect of assignment to intervention)</td><td>Some concerns of risk of bias</td><td>Quote: “Due to the lack of a placebo device, clinical, and research staff were not blinded to treatment allocation.”

Quote: “[Protocol adherence] Tracking logs were retrieved from the medical charts of 59 participants at study completion.”

Due to this large portion of missing information about protocol adherence and implementation of the intervention, and how this may have differed between groups, review authors do not have enough information to be able to ascertain whether there were any deviations from the intended protocol that may have arisen from the unblinded nature of the trial.</td></tr>
<tr><td colspan="2">Domain 3: Risk of bias due to missing outcome data</td><td>Low</td><td>Data from all participants in each group is available for the colonization outcome, as shown in Table 3 of Pook et al.</td></tr>
<tr><td>Outcome 3: Colonization</td><td>Domain 4: Risk of bias in measurement of the outcome</td><td>Low</td><td>Quote: “We will define central line colonization as a positive central line culture with concurrent negative peripheral stab culture. We will not order peripheral stab cultures to avoid harm, but will collect data from those ordered by the treating team.”

Quote: “A single set of blood cultures will be drawn from the CVC of all study participants every 48 h beginning on the day of study enrolment until the patient has all CVCs removed, has a documented infection, or leaves the ICU. Blood cultures will be analyzed for infectious organisms in the core laboratory […].”

There is no evidence to suggest outcome definition used or method of measurement was inappropriate. There is no evidence to suggest outcome measurement different between treatment groups.

Study authors report similar numbers of samples collected between groups, as well as no significant differences between ICU length-of-stay between groups.

Review of supplemental materials associated with the previously-published study protocol also indicate that blinding was maintained by laboratory</td></tr>
</table>

			personnel during their analysis of CVAD samples, as the data inputted into the electronic medical record system did not include any information about randomization status of the patient.
	**Domain 5:** Risk of bias in selection of the reported result	Low	All results were reported in accordance with what was presented in the previously published protocol
	**Overall Risk of Bias: Some concerns of risk of bias**		

Parienti et al., 2014

Sources Reviewed:
In order to complete this quality appraisal, review authors reviewed the original study article, and related article containing details of the study from where the control cohort was sourced (Parienti et al., 2008). We also connected with study authors regarding clarification of any deviations from the intervention as well as handling of missing data. Study authors replied with all information necessary.

**Domain:**	**Judgement:**	**Support from text of the paper:**
**Domain 1:** Bias due to confounding	Serious	Quote: "We used propensity-score methods, namely, marginal structural models (MSM) and 2:1 greedy propensity-score matching (PSM), to ensure that the Cathedia and CLocks populations were comparable."<br><br>Quote: "In addition, MSM was used in a sensitivity analysis by restricting the Cathedia cohort to the patients admitted to the Caen University hospital only (the same institution as in the CLock cohort). We considered that a 10% change in the effect size for the primary endpoint would denote significant confounding compared to the full Cathedia sample."<br><br>Quote: "The standardized difference between groups was less than 10% for all baseline characteristics after propensity-score matching."<br>Authors adjusted for effect caused by differences in baseline characteristics and risk factors between groups<br><br>There is sufficient evidence from the text of the paper to suggest that confounding due to multiple risk factors of developing CVAD complication was adequately controlled for (example: age, BMI, severity of illness, antibiotic use, immune status, presence of various comorbidities).<br><br>Unfortunately, other standard CVAD care procedures was not standardized between the past control cohort and the prospectively recruited intervention group:<br><br>Quote: "Between the two time periods (2004 to 2007 for the Cathedia historical group and 2011 to 2012 for the CLock group), the effectiveness of the implementation of simple "bundle measures" to prevent the risk of CRBSI in patients admitted to intensive care units could favor the CLock group, independently of the locking solution used. Maximal barrier precautions for catheter insertion and maintenance were already in use during the control period, chlorhexidine skin antisepsis was not available, avoidance of the femoral vein for vascular access was not relevant compared to avoidance of the jugular vein and exposures to central venous access were similar between the two periods. Moreover, the patient risk factors and interventions that could increase or decrease the risk of catheter infection were prospectively monitored and standardized.

		Nevertheless, the possible increased use of ultrasound for catheter insertion from 2004 and 2012, as well as other unrecorded confounding factors, has not been taken into account."<br><br>The seminal study conducted by the Michigan Health and Hospital Association in 2003 resulted in widespread adaptation of the 'Keystone Bundle Intervention' prior to CVAD insertion. These changes were incorporated into the CDC guideline for the prevention of intravascular catheter-associated bloodstream infections in 2011. As such, risk of bias due to time is a consideration for this paper.
**Domain 2:** Bias in selection of participants into the study	Low	Quote: "To serve as controls, we used the patients included in the Cathedia study, which was a multicenter study involving the Caen University Hospital in addition to 8 university hospitals and 3 general hospitals. The comparison of a group of patients admitted to a single institution with a group of patients admitted to the same institution plus 11 other institutions cannot adjust for a possible center effect. Therefore, we also conducted a sensitivity analysis by restricting the control group to patients admitted to the Caen University hospital."<br><br>A possible selection bias exists due to the Cathedia control group being originally recruited from multiple hospitals. Parienti et al. addressed this by performing the 2:1 propensity matching to select a control group that includes only Cathedia participants from the same hospital as the CLock intervention group. This introduces a potential post-intervention selection bias in the control group. However, as all hospitals included in the Cathedia study were in the same country and adhere to the same CVAD care guidelines, there is little to suggest this variable would have a large effect on outcome measures. Additionally, following the 2:1 PSM techniques described, this new control group was found to have no statistically different baseline differences in relevant risk factors that may lead to CVAD complication, as seen in Parienti et al., Table 1. Participants were followed for the same amount of time in both control and intervention cohorts. As such, we adjudicate the risk of selection bias to be low.
**Domain 3:** Bias in classification of interventions	Low	Intervention groups were clearly defined, and the type, dose, and frequency of locking solution is well defined for both groups. Additionally, the citrate group was recruited four years following the control group. As such review authors rate the potential for misclassification to be low.
**Domain 4:** Bias due to deviations from intended interventions	Low	There is no information presented in the papers regarding deviations from the study protocol, however there is little evidence to suggest that knowledge of the intervention would cause deviation in study procedures. After contacting the corresponding author of the study, we confirmed that all patients received the intended intervention without deviation.

Outcome 1: CRBSI	**Domain 5:** Bias due to missing data	No Information	Correspondence with study authors revealed that 15% of the catheters were not sent to culture in the control cohort. This missing data was remedied by excluding these patients and performing a modified intention-to-treat analysis. In the intervention cohort, there was an unknown number of catheters not sent for analysis. The study authors presented a complete case analysis, without accounting for any missing data.<br>As an unknown amount of data is missing from the intervention cohort, review authors do not have enough information to assess whether the amount of missing data was comparable between study groups and if reasons for missing data were comparable between study groups.
	**Domain 6:** Bias in measurement of outcomes	Low	Quote: "Data were prospectively collected as a part of the French REA-Raisin surveillance network, which targets device-associated infections."<br><br>Quote: "In the Cathedia and CLock cohorts, all dialysis catheters were aseptically removed and systematically sent to culture, regardless of the clinical patients' statuses."<br><br>Quote: "CRBSI was defined as catheter-tip colonization with one concordant peripheral blood culture for pathogens and two concordant peripheral blood cultures for potential skin contaminants.<br><br>Due to standardized microbiological testing techniques used, there is no evidence that any knowledge of the intervention group would influence the outcome measures of CRBSI. It is likely that CRBSI was assessed as part of a larger surveillance program that involved assessors that were blinded to locking fluid used.
	**Domain 7:** Bias in selection of reported result	Low	There is clear evidence from examination of the pre-specified analysis plan that the results of all analysis were presented in the final publication. Results from both subgroups (before and after matching) are reported in the paper.
	**Overall Risk of Bias: Serious**		
Outcome 2: Occlusion	**Domain 5:** Bias due to missing data	No Information	Correspondence with the study authors confirmed data was reasonably complete, however the difference in quantity of missing data between groups and reason for missing data is unknown. Not enough information is present to adjudicate this outcome.
	**Domain 6:** Bias in measurement of outcomes	Low	Quote: "Catheter dysfunction, which served as the secondary endpoint in the Cathedia trial, was similarly defined as an inability to attain an adequate blood flow, requiring catheter replacement."<br><br>Correspondace with study authors confirm that all hemodialysis catheters underwent standard care throughout both study periods, which includes regular evaluation of catheter patency. It is unlikely that any knowledge of

			intervention resulted in bias of outcome measurements, due to the objective nature of patency assessment.
	**Domain 7:** Bias in selection of reported result	Low	There is clear evidence from examination of the pre-specified analysis plan that the results of all analysis were presented in the final publication. Results from both subgroups (before and after matching) are reported in the paper.
	**Overall Risk of Bias: Serious**		
Outcome 3: Colonization	**Domain 5:** Bias due to missing data	No Information	Correspondence with study authors revealed that 15% of the catheters were not sent to culture in the control cohort. This missing data was remedied by excluding these patients and performing a modified intention-to-treat analysis. In the intervention cohort, there was an unknown number of catheters not sent for analysis. The study authors presented a complete case analysis, without accounting for any missing data.<br>As an unknown amount of data is missing from the intervention cohort, review authors do not have enough information to assess whether the amount of missing data was comparable between study groups and if reasons for missing data were comparable between study groups.
	**Domain 6:** Bias in measurement of outcomes	Low	Quote: “Data were prospectively collected as a part of the French REA-Raisin surveillance network, which targets device-associated infections.”<br><br>Quote: “In the Cathedia and CLock cohorts, all dialysis catheters were aseptically removed and systematically sent to culture, regardless of the clinical patients’ statuses.”<br>Quote: “The primary endpoint of this study was catheter-tip colonization, defined as cultures with greater than or equal to 10^3 CFU per millimeter of growth, according to the Brun-Buisson vortex technique without neutralizing broth.”<br><br>Due to standardized microbiological testing techniques used, there is no evidence that any knowledge of the intervention group would influence outcome measurement.
	**Domain 7:** Bias in selection of reported result	Low	There is clear evidence from examination of the pre-specified analysis plan that the results of all analysis were presented in the final publication. Results from both subgroups (before and after matching) are reported in the paper.
	**Overall Risk of Bias: Serious**		
